# Scalable single-cell RNA sequencing from full transcripts with Smart-seq3xpress

**DOI:** 10.1038/s41587-022-01311-4

**Published:** 2022-05-30

**Authors:** Michael Hagemann-Jensen, Christoph Ziegenhain, Rickard Sandberg

**Affiliations:** Department of Cell and Molecular Biology, Karolinska Institutet, Stockholm, Sweden

**Keywords:** Biological techniques, Biotechnology

## Abstract

Current single-cell RNA sequencing (scRNA-seq) methods with high cellular throughputs sacrifice full-transcript coverage and often sensitivity. Here we describe Smart-seq3xpress, which miniaturizes and streamlines the Smart-seq3 protocol to substantially reduce reagent use and increase cellular throughput. Smart-seq3xpress analysis of peripheral blood mononuclear cells resulted in a granular atlas complete with common and rare cell types. Compared with droplet-based single-cell RNA sequencing that sequences RNA ends, the additional full-transcript coverage revealed cell-type-associated isoform variation.

## Abstract (web summary)

Smart-seq3xpress enables single-cell RNA sequencing from full transcripts at high throughput.

## Main

Many single-cell RNA sequencing (scRNA-seq) methods have been described and their respective strengths and weaknesses characterized^1^. However, researchers still face a compromise between methods with high cell throughput (that is, droplet or combinatorial indexing methods) but low transcript coverage and methods with high sensitivity and full-length transcript coverage (that is, plate-based methods). Based on Smart-seq3 (ref. ^2^)—the method currently offering the highest information content per profiled cell—we systematically evaluated the feasibility of reducing volumes, reagents and experimental steps, without sacrificing data quality. This resulted in Smart-seq3xpress, a scalable nanoliter implementation of Smart-seq3 with throughput limited only by the available equipment (for example, polymerase chain reaction (PCR) instruments), and sequencing-ready libraries can be generated in a single workday.

**Fig. 1:**
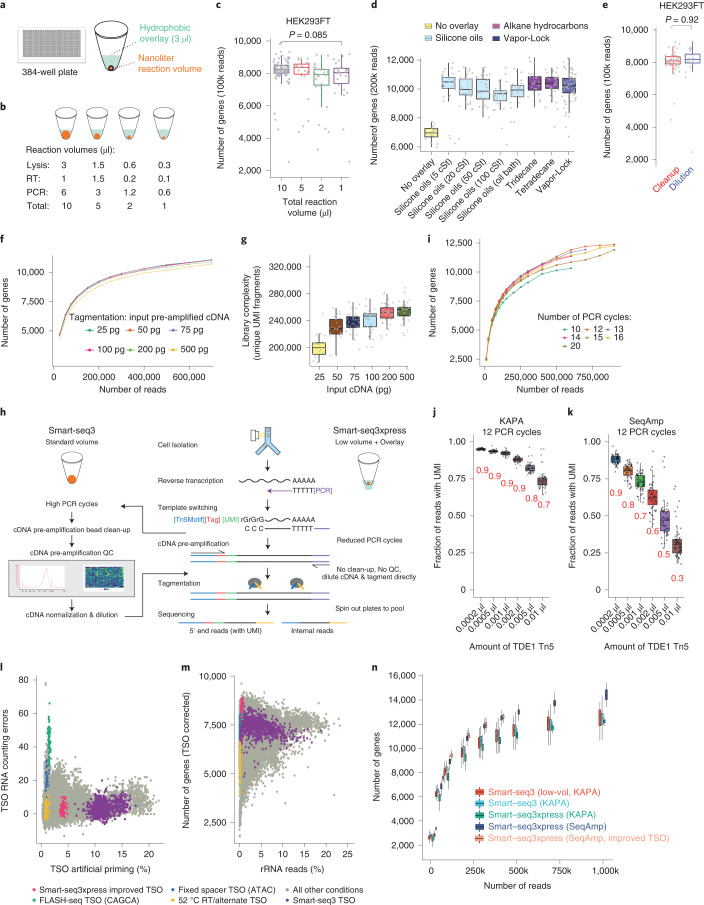
Scalable full-transcript coverage scRNA-seq with Smart-seq3xpress. **a**, Schematic of nanoliter cDNA synthesis reactions performed in wells of 384-well PCR plates with 3 µl of hydrophobic overlay. **b**, Illustration of reduced-volume experiments with the lysis, RT and PCR volumes used. **c**, The number of genes detected per HEK293TF cell at each reaction volume, when sampling 100,000 sequencing reads (*n* = 100, 19, 32 and 28 cells, respectively). *P* value represents a two-sided *t*-test between the 10-µl and 1-µl conditions. **d**, Influence of hydrophobic overlays on miniaturized cDNA synthesis (1 µl total volume). For each compound, boxes depict the number of genes detected per HEK293FT cell (*n* = 17, 34, 39, 28, 25, 24, 28, 38 and 70, respectively), subsampled at 200,000 sequencing reads per cell. **e**, Replacement of the bead-based cDNA cleanup by dilution in single HEK293FT (*n* = 58 and 52, respectively) cells. Box plots show the number of genes detected per cell and condition (at 100,000 reads) with *P* value for a two-sided *t*-test across conditions. **f**, Tagmentation complexity using 0.1 µl of ATM Tn5 enzyme per HEK293FT cell in relation to input cDNA. The median number of detected genes as a function of raw sequencing reads (*n* = 51, 53, 54, 53, 53 and 52 cells for 25, 50, 75, 100, 200 and 500 pg, respectively). **g**, Tagmentation complexity for varying amounts of cDNA input. Complexity was summarized as unique aligned and gene-assigned UMI-containing read pairs per 400,000 raw reads and HEK293FT cell (*n* = 49, 51, 51, 50, 51 and 44). **h**, Schematic outline of the Smart-seq3 and Smartseq3xpress workflows. **i**, The number of genes detected with Smart-seq3xpress after variable amounts of pre-amplification PCR cycles. Median number of genes is reported as a function of raw sequencing reads in HEK293FT cells (*n* = 93, 98, 108, 113, 102, 114 and 118 cells for 10, 12, 13, 14, 15, 16 or 20 cycles, respectively). **j**, Fraction of UMI-containing reads to internal reads for HEK293FT cells prepared with Smartseq3xpress (KAPA HiFi; 12 PCR cycles), at a variable range of TDE1 Tn5 amounts (*n* = 64 cells each). **k**, Fraction of UMI-containing reads to internal reads for HEK293FT cells prepared with Smartseq3xpress (SeqAmp; 12 PCR cycles), at a variable range of TDE1 Tn5 amounts (*n* = 60 cells each). **l**,**m**, Optimization of RT and PCR conditions across 376 experimental conditions on HEK293FT cells. Colors indicate particular experimental conditions: Smart-seq3xpress with Smart-seq3 TSO (purple; *n* = 912), 52 °C RT/alternate TSO implementation (yellow; *n* = 74), fixed spacer TSO variant (blue; *n* = 45), FLASH-seq TSO variant (green; *n* = 55), Smart-seq3xpress with improved TSO (pink; *n* = 63) and all other conditions (gray; *n* = 21,707). Scatter plots denote the level of artifactual TSO-UMI reads and RNA counting errors (**l**) as well as a percentage of ribosomal RNA (rRNA) mapped reads and number of detected genes in 100,000 reads after removal of strand invasion reads (**m**). **n**, Benchmarking of Smart-seq3 variants. Box plots show the number of genes detected per HEK293FT cell in full-volume Smart-seq3 (ref. ^2^), low-volume Smart-seq3 and Smart-seq3xpress implementations, at the indicated read depths (*n* = 109–110, 18–27, 9–170, 20–55 and 9–63 cells, depending on the cells available at the given sequencing depths). The box plots (in **c**, **d**, **e**, **j**, **k** and **n**) show the median and first and third quartiles as a box, and the whiskers indicate the most extreme data points within 1.5 lengths of the box. cSt, centistoke.

**Extended Data Fig. 1:**
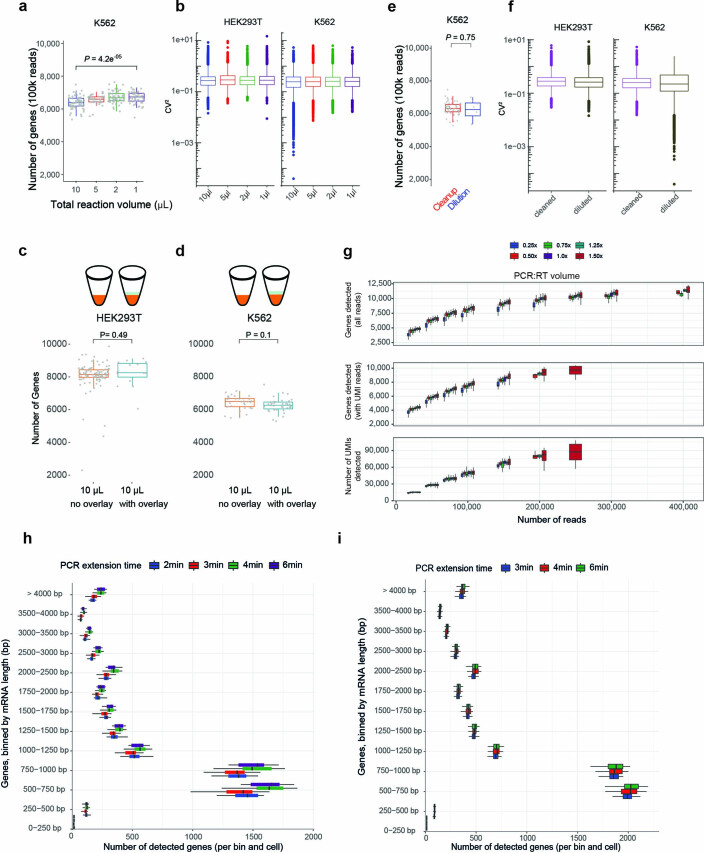
Optimization of low-volume cDNA synthesis. **(a)** Reduction of reaction volumes in single K562 cells. Shown are the number of genes detected per cell at each reaction volume when sampling 100,000 sequencing reads (n = 63, 39, 55, 53 cells, respectively). P-value represents a t test between the 10 µL and 1 µL conditions. (**b**) Coefficient of variance of cells at scaled volumes in both HEK293FT (n = 100, 19, 32, 28 cells, respectively) and K562 cell lines (n = 63, 39, 55, 53 cells, respectively). (**c**-**d**) For both (**c**) HEK293FT and (**d**) K562 cells the influence of creating standard volume Smart-seq3 libraries without (HEK293FT n = 37; K562 n = 26) and with an overlay (VaporLock; HEK293FT n = 15; K562 n = 31) was compared. Boxplots show genes detected and p-values show result of a two-sided t-test. (**e**) Replacement of the bead-based cDNA cleanup by dilution in single K562 (n = 57, 38, respectively) cells. Shown are the number of genes detected per cell for each condition at 100,000 reads with a p-value for a two-sided *t*-test within cell types. (**f**) Coefficient of variance between cells having received cDNA clean-up or dilution in HEK293FT (n = 58, 52, respectively) and K562 (n = 57, 38, respectively). (**g**) For indicated ratios of PCR volume to RT volume, boxplots show genes detected, genes with UMIs detected and UMIs captured, downsampled by sequenced reads. For PCR ratios 0.1x (n = 43), 0.2x (n = 48), 0.3x (n = 48), 0.4x (n = 47), 0.5x (n = 47), 0.6x (n = 45) HEK293FT cells were analyzed. (**h**) Boxplots for each PCR extension time (2, 3, 4, 6 min) using KAPA HiFi Hot Start polymerase are shown as detected genes binned by their transcript length at 250,000 reads per cell. (n = 29, 24, 29, 10; respectively) (**i**) Number of genes detected binned by transcript length for each extension time 3 min (n = 64), 4 min (n = 64), 6 min (n = 60) using SeqAmp polymerase at 250,000 reads per cell. The boxplots (in **a-i**) show the median, first and third quartiles as a box, and the whiskers show the most extreme data points within 1.5 lengths of the box.

We hypothesized that the Smart-seq3 chemistry would work in substantially lower volumes when covered in an inert hydrophobic substance (‘overlay’) (Fig. 1a). Using accurate non-contact nanoliter dispensers, we scaled the reaction volumes of the lysis, reverse transcription (RT) and pre-amplification PCR steps down to 1:2, 1:5 and 1:10 of the established volumes and tested these conditions on K562 and HEK293FT cells (Fig. 1b). For the lowest volumes, cells were sorted by fluorescence-activated cell sorting (FACS) into 300 nl of lysis buffer covered with 3 µl of Vapor-Lock, with subsequent additions of 100 nl for RT and 600 nl for PCR. After shallow sequencing, alignment and error correction of reads, we observed similar numbers of detected genes and molecules per cell at a sequencing depth of 100,000 reads per cell (Fig. 1c and Extended Data Fig. 1a,b), confirming that reaction scaling is possible without compromising data quality or introducing unwanted variability. Further reduction of reaction volumes beyond 1:10 was also possible, although not further pursued as savings in reagents are diminishing and reactions may become vulnerable to variations in cell sorting fluid (~5 nl). We hypothesized that the overlays would both protect the low reaction volumes from evaporation and provide a ‘landing cushion’ for the FACS-sorted cells. Indeed, many overlays with varying chemical properties could be used with low-volume Smart-seq3 (Fig. 1d), including silicone oils with high viscosities and hydrocarbons with higher freeze points. As expected, overlays did not interfere with the cDNA synthesis reaction when tested in larger volumes (Extended Data Fig. 1c,d). Performing low-volume Smart-seq3 without an overlay resulted in drastic losses (Fig. 1d), explaining why earlier efforts to miniaturize plate-based scRNA-seq without overlay resulted in substantially decreased complexity^3–5^. Next, we investigated whether the time-consuming and plastics-consuming cDNA clean-up step could be omitted by instead diluting the cDNA now obtained in lower volumes. At equal sequencing depths, single-cell libraries generated with cDNA dilution and clean-up had no detectable differences (*P* = 0.89 and *P* = 0.71, respectively, *t*-test; Fig. 1e and Extended Data Fig. 1e,f). The lowered reaction volumes enabled better control of downstream reaction conditions, and we, therefore, investigated the relationship between RT and pre-amplification PCR volumes. Dilution of the RT reaction was found beneficial for optimal PCR performance, with the established 1.5× ratio of PCR to RT volume (Extended Data Fig. 1g). However, PCR extension times could be reduced from 6 minutes to 4 minutes without complexity losses for longer transcripts (Extended Data Fig. 1h–j).

**Extended Data Fig. 2:**
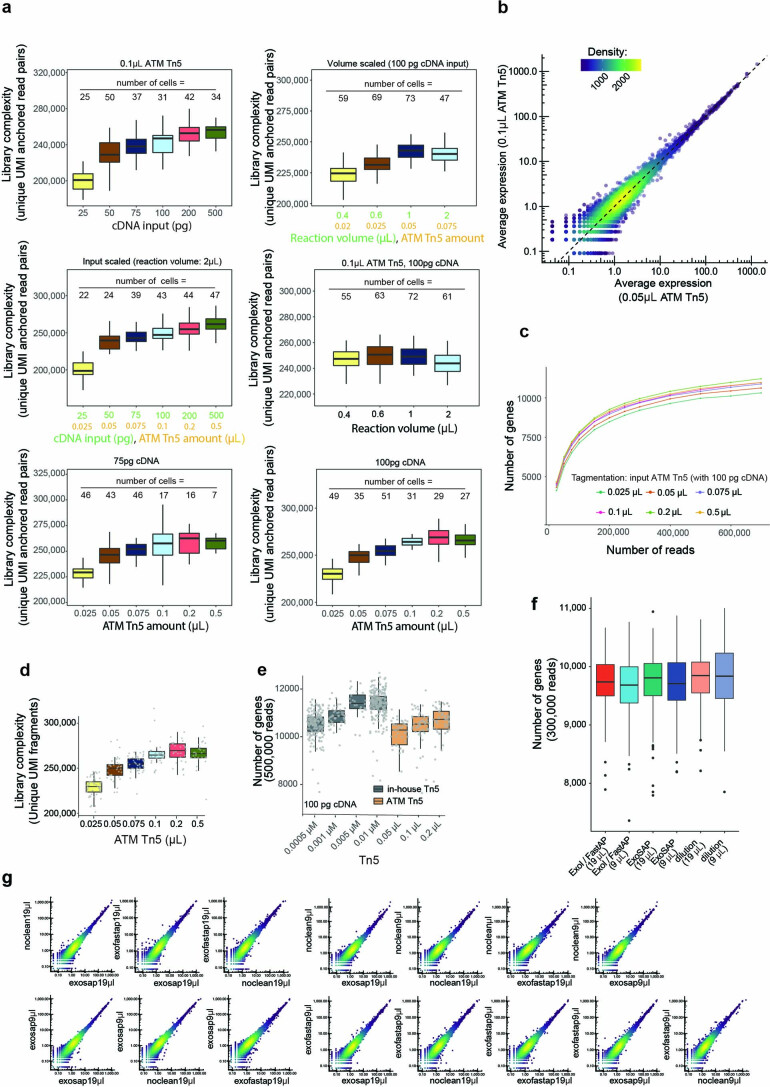
Investigating tagmentation complexity. (**a**) Systematic investigation of tagmentation complexity was performed by varying cDNA input with constant Tn5 amount, varying input and Tn5 amounts, varying Tn5 amounts with constant cDNA input, scaling of reaction volumes and Tn5 amounts with constant cDNA input, scaling of reaction volumes with constant cDNA and Tn5 amounts. For each boxplot, shown is the library complexity in terms of unique gene-assigned UMI-anchored read pairs (unique per-molecule cut-sites) from 400,000 raw sequencing reads. Each condition contains between 22 and 73 HEK293FT cells as annotated above each box. (**b**) Concordance of gene expression levels between HEK293FT cells tagmented using 0.05 µL ATM Tn5 (n = 23 cells) and 0.1 µL ATM Tn5 (n = 11 cells) (mean UMI counts over 15,915 genes). (**c**) Tagmentation complexity using 100 pg cDNA per single HEK293FT cell in relation to the enzyme amount (ATM Tn5). For each dot, the median number of detected genes is calculated from the indicated number of raw sequencing reads and plotted from n = 55, 54, 56, 58, 52, 51 cells (0.025 µL, 0.05 µL, 0.075 µL, 0.1 µL, 0.2 µL, 0.5 µL). (**d**) For varying amounts of Tn5 enzyme (see (c)), tagmentation complexity was summarized as unique aligned and gene-assigned UMI-containing read-pairs per 400,000 raw reads per HEK293FT cell (n = 53, 46, 56, 57, 52, 50, respectively). (**e**) The use of in-house Tn5 relative to performance of ATM Tn5 was compared in HEK293FT cells. Each boxplot shows the number of genes detected at 500,000 raw reads (n = 182, 52, 24, 226, 41, 53, 50, respectively). (**f**) Influence of post PCR clean-up for Smartseq3xpress. Genes detected after treating preamplified cDNA libraries to reduce “contaminants”, such as dNTPs and oligonucleotides, before going into tagmentation. All conditions (dilution alone (n = 152, 129), ExoSAP IT-express (“ExoSAP”; n = 254, 178), or ExoI + Fast-AP (n = 259, 167)) were either diluted in 9 or 19 µL of water. (**g**) Influence of post PCR clean-up for UMI captures. Pairwise comparisons of mean expression estimates (mean UMIs per gene) for evaluated clean-up conditions. The boxplots shown in **a**, **d**, **e** and **f** indicate the median, first and third quartiles as a box, and the whiskers show the most extreme data point within 1.5 lengths of the box.

A major cost in plate-based scRNA-seq is tagmentation, which needs to be performed individually on each cell, and, although reducing the amounts of commercial Tn5 has been suggested to cut reaction costs^6,7^, it is currently unclear to what degree Tn5 can be reduced without losing library complexity. We investigated how the cDNA input amounts influence library complexity, which revealed that 20-fold variations in cDNA amounts could be tolerated with minor effects on gene and unique fragment detection (Fig. 1f,g). Library complexity was mainly a product of absolute Tn5 and cDNA amounts with little effect from varying reaction volumes (Extended Data Fig. 2a,b). The slightly reduced complexity at the highest cDNA amounts (Fig. 1f) likely resulted from insufficient tagmentation due to the limited Tn5 amounts used. Similar results were observed when varying the Tn5 amounts on a fixed amount of cDNA input (Extended Data Fig. 2c,d), and high-quality libraries were obtained using both commercial amplicon tagmentation mix (ATM) Tn5 and in-house^8^ Tn5 (Extended Data Fig. 2e). Altogether, these results demonstrated that tagmentation reactions were robust over large variations in cDNA input and Tn5 amounts and that considerable savings in resources can be made in reducing Tn5 amounts with only minimal effect on library complexity (Extended Data Fig. 2d).

**Extended Data Fig. 3:**
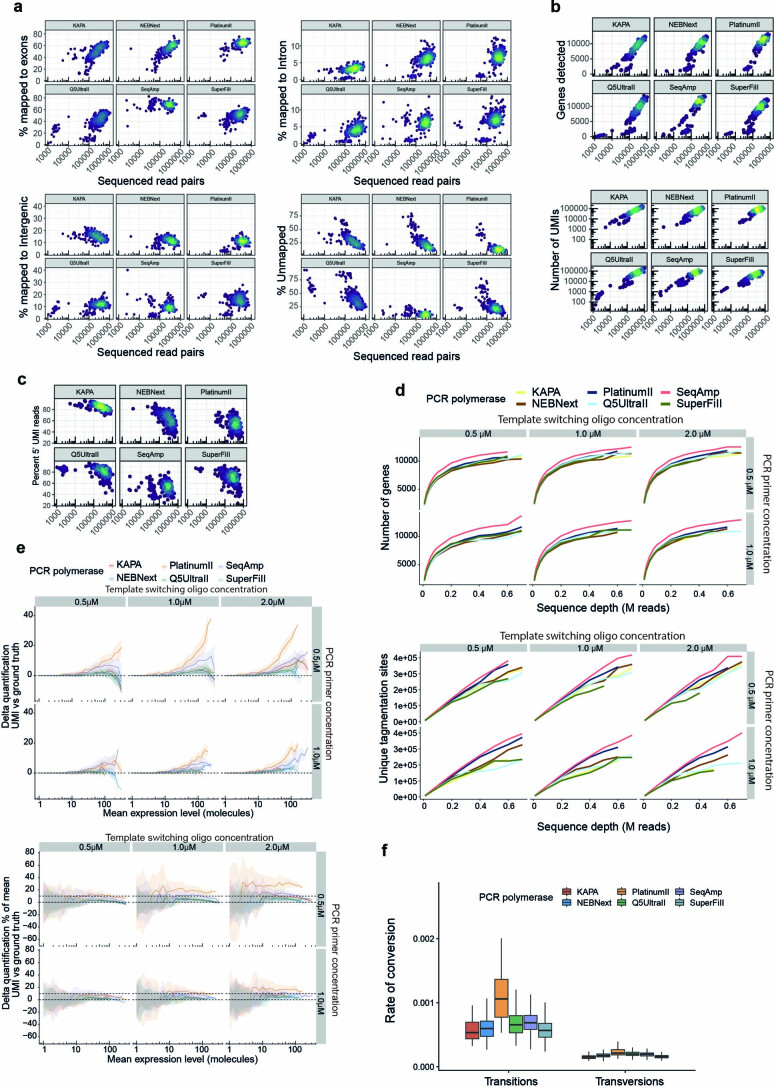
Performance of pre-amplification polymerases for Smartseq3xpress. (**a**) Mapping statistics of the six different tested commercial polymerases: KAPA HiFi Hot Start (n = 384), NEBNext Q5 (“NEBNext”, n = 384), NEBNext Ultra II Q5 (“Q5UltraII”, n = 384), Platinum II (n = 384), SuperFi II (n = 384), SeqAmp (n = 360). Dotplots show percentage of mapped reads to exons, introns, intergenic regions and unmapped with the sequenced read depth. (**b**) Number of Genes and UMIs detected per cell relative to sequencing depth for all six polymerases tested. (**c**) Fraction of UMI-containing reads versus internal reads for each of the tested polymerases. (**d**) Lines show median number of genes detected and median number of unique tagmentation sites after downsampling of sequenced reads using 0.5 µM, 1.0 µM and 2.0 µM TSO concentration in RT, combined with either 0.5 µM or 1 µM of each forward and reverse PCR primer. The replicate numbers for each of the panels are: TSO = 0.5 µM, PCR = 0.5 µM: n = 58, 57, 62, 63, 52, 55 cells; TSO = 0.5 µM, PCR = 1.0 µM: n = 51, 60, 61, 45, 51, 55 cells; TSO = 1.0 µM, PCR = 0.5 µM: n = 57, 58, 61, 52, 51, 57 cells; TSO = 1.0 µM, PCR = 1.0 µM: n = 51, 56, 63, 49, 52, 55 cells; TSO = 2.0 µM, PCR = 0.5 µM: n = 62, 59, 61, 53, 54, 51 cells; TSO = 2.0 µM, PCR = 1.0 µM: n = 49, 53, 59, 50, 55, 49 cells (KAPA, PlatinumII, SeqAmp, NEBNext, Q5UltraII, SuperFiII, respectively). (**e**) Molecular spike-ins were used to assess the accuracy of each polymerase in mRNA molecule counting, based on the counting difference between internal molecular spikes counts and Smart-seq3xpress UMIs at indicated amounts of TSO and PCR primer concentrations. Colored lines indicate the mean counting difference between the unique spike identifiers and quantified UMIs when sampling the spike at the indicated mean expression levels for each of the polymerases, shaded by the standard deviation. The counting differences are expressed in absolute deviance or relative to the mean molecule number. (**f**) Rate of base conversions in aligned reads relative to the reference genome. For every polymerase, we compute the average fraction of transitions and transversions, shown as boxplots over all cells, n = 328, 367, 315, 343, 302, 322 (for KAPA, PlatinumII, SeqAmp, NEBNext, Q5UltraII, SuperFiII). Boxplots indicate the median, first and third quartiles as a box, and the whiskers show the most extreme data point within 1.5 lengths of the box.

Having demonstrated robust tagmentation over large ranges of cDNA, Tn5 and reaction volumes, we realized an opportunity to exclude several time-consuming and resource-intensive experimental steps, including excessive cDNA pre-amplification, concentration measurements, fragment length quality control (QC) traces and cDNA amount normalization (Fig. 1h). Instead, cDNA products from fewer pre-amplification cycles could be directly tagmented without the above-mentioned steps—this strategy we termed Smart-seq3xpress. To explore this potential, we first generated libraries with low-volume RT (300 nl lysis volume) from HEK293FT cells and using a range of PCR pre-amplification cycles (10–20), which revealed very similar gene detection (Fig. 1i) without any need for additional enzymatic reaction clean-ups (Extended Data Fig. 2f,g). However, the resulting libraries were heavily biased toward 5′ reads that contain the unique molecular identifier (UMI) at the expense of the internal reads important for full-transcript coverage scRNA-seq^2^. This resulted from inefficient tagmentation and the inability to modulate the ratio of UMI-containing and internal reads by Tn5 amounts (Fig. 1j). Specifically, the high salt concentration in the KAPA PCR buffer likely resulted in template blocking during tagmentation^9^, whereas other PCR polymerases were compatible with direct tagmentation (Extended Data Fig. 3a,b). Notably, tagmentation of SeqAmp and Platinum II amplified cDNA had considerably lowered fraction of 5′ UMI reads, indicative of improved tagmentation compatibility (Extended Data Fig. 3c). Additional experiments identified SeqAmp as the preferred PCR polymerase, as it consistently performed well with improved gene and molecule detection over a range of template-switching oligonucleotide (TSO) and PCR primer amounts (Extended Data Fig. 3a–d).

To directly assess the accuracy of RNA counting in Smart-seq3xpress, we used the newly developed molecular spikes^10^ (UMIcountR R package). Whereas Platinum II had higher error rates, which resulted in inflated RNA counts (Extended Data Fig. 3e,f), SeqAmp with 2 µM TSO and 1 µM of each PCR primer had high sensitivity and accuracy, and the fraction 5′ UMI reads could be modulated as expected by varying the cDNA or Tn5 amounts (Fig. 1k).

**Extended Data Fig. 4:**
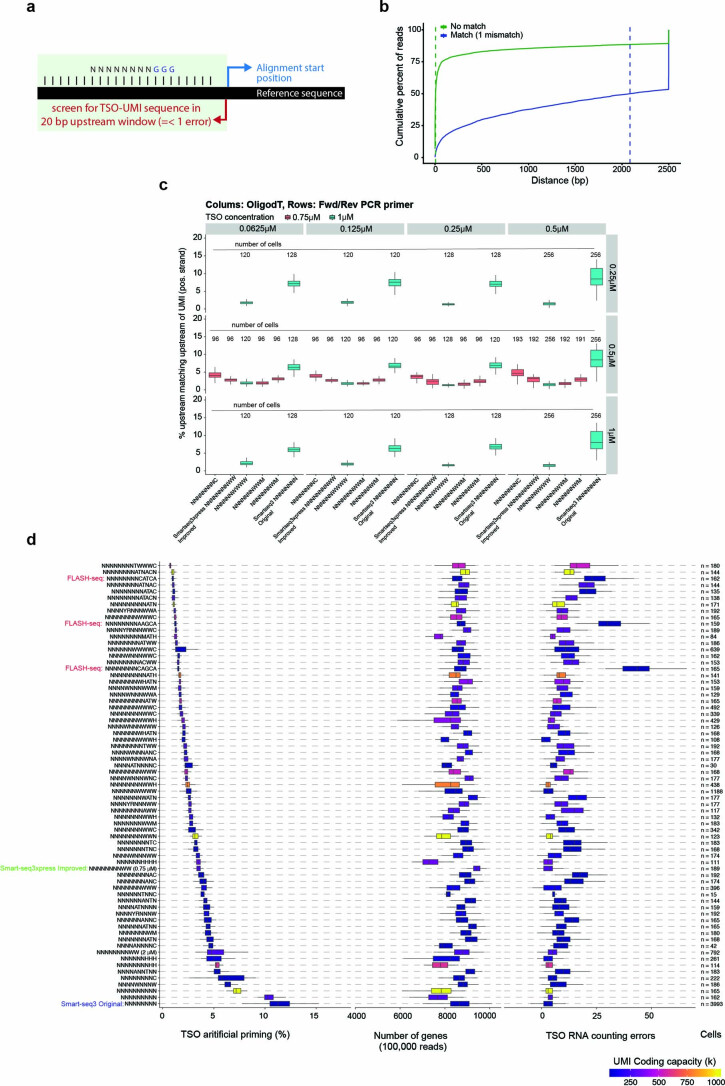
TSO strand-invasion artifact and improved TSO designs. (**a**) Schematic representation of the search procedure for artifactual TSO-UMI reads. For every aligned 5’ read, a 20 bp window of reference genome sequence upstream of the alignment start position is considered. Within this sequence, we search for the UMI sequence allowing up to 1 mismatch. (**b**) Strand invasion leads to shortened captured molecules. We grouped reads by the presence or absence of TSO-UMI match in the 20 bp upstream window and retrieved the closest annotated transcription start site (TSS) of the assigned gene. Shown is the distance to TSS as the cumulative percentage of reads analyzed. (**c**) Influence of TSO design (x-axis), oligo-dT primer amount (columns) and forward/reverse PCR primer amounts (rows) on the occurrence of strand-invasion artifacts (y-axis). TSO concentration is indicated in color (red: 0.75 uM, teal: 1 µM). Replication was performed over many cells per condition as annotated above each respective box. (**d**) Shown are all evaluated UMI sequences incorporated into the Smart-seq3 TSO with their base composition in terms of random or stable bases. For each TSO, we show sequencing metrics in HEK293FT cells (numbers of cells per condition annotated in the Figure), in terms of the frequency of artificial TSO priming, Number of genes detected after discarding TSO primed molecules (100,000 raw reads) and the accuracy of the UMI counting as assayed by molecular spikes. Every box is colored by the coding capacity of the associated random bases in the TSO. Boxplots in **c** and **d** indicate the median, first and third quartiles as a box, and the whiskers show the most extreme data point within 1.5 lengths of the box.

**Extended Data Fig. 5:**
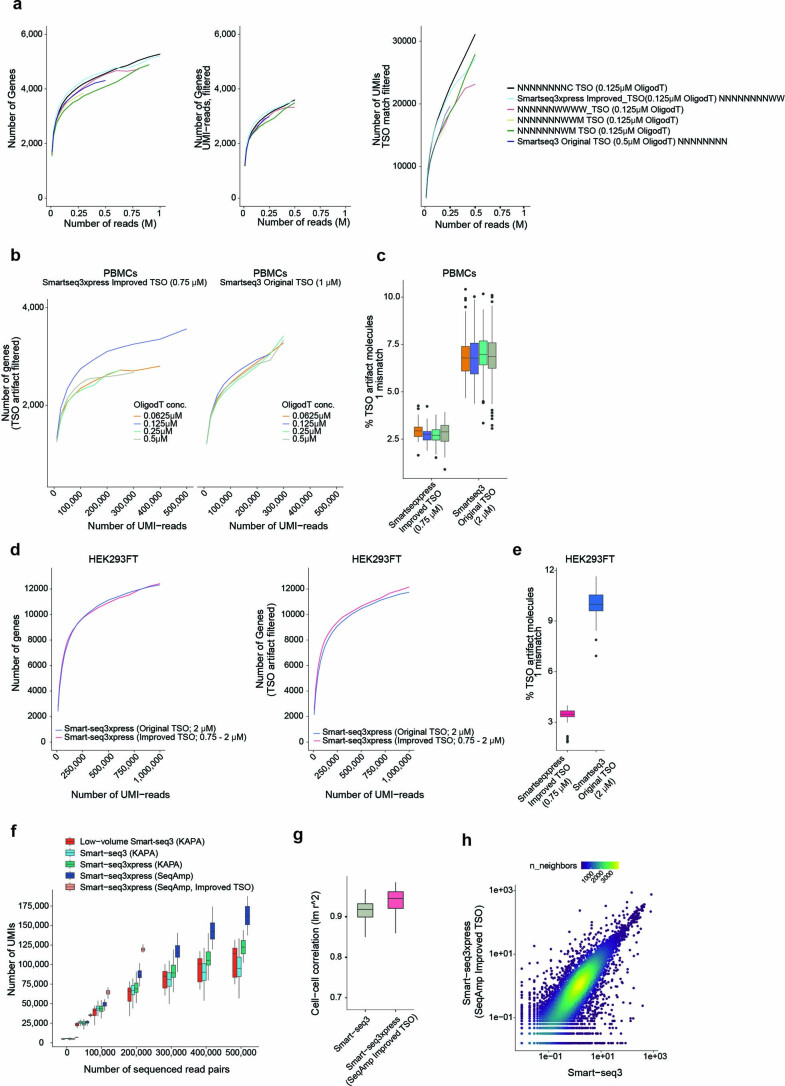
Evaluation of candidate reaction conditions in human PBMCs and HEK293FT cells. (**a**) Candidate TSO sequences and previous TSO (“Smartseq3 Original”) were evaluated in human PBMC samples with a change to 0.125 µM oligo-dT primer. At indicated sequencing depths, we show the median number of detected genes (left), median number of detected genes after discarding TSO-priming artifact reads (middle), and median number of detected UMIs after discarding TSO-priming artifact reads (right). (**b**) Investigation of the optimal amount of oligo-dT primer (colors) in the context of new TSO (“Smartseq3xpress improved”; left; n = 74, 71, 74, 83 cells for 0.0625 µM, 0.125 µM, 0.25 µM, 0.5 µM, respectively) and previous TSO (“Smartseq3 Original”; right; n = 293, 286, 313, 319 cells for 0.0625 µM, 0.125 µM, 0.25 µM, 0.5 µM, respectively) in PBMCs. Shown are the median number of detected genes per cell after discarding TSO-priming artifact reads. (**c**) Frequency of TSO artifact reads in PBMC cells for new TSO (“Smartseq3xpress Improved”; left; n = 64, 63, 53, 58 cells, respectively) and previous TSO (“Smartseq3 Original”; right; n = 266, 256, 262, 251 cells, respectively), with colors denoting the amount of oligo-dT primer used. (**d**) Benchmarking of new Smart-seq3xpress Improved TSO in HEK293FT cells. At the indicated sequencing depth, we show the number of genes in internal+UMI reads (left) and TSO-artifact filtered UMI reads (middle). (**e**) The reduction in the occurrence of TSO primed strand-invasion artifacts is shown as a boxplot for n = 94, 330 HEK293FT cells (improved TSO, original TSO). (**f**) Benchmarking of Smartseq3 variants and Smart-seq3xpress iterations. Shown are the number of UMIs captured in HEK293FT cells in the full-volume Smart-seq3 (n = 110 cells), low-volume Smart-seq3 (n = 27 cells) and Smart-seq3xpress iterations (n = 170, 55, 63 cells for KAPA, SeqAmp and SeqAmp improved TSO, respectively) at the indicated read depths. (**g**) Reproducibility over cells visualized by cell-to-cell correlation for Smartseq3 and Smartseq3xpress (n = 107, 62, respectively). Two-sided *t*-test p-value < 2.2e-16. (**h**) Representative correlation in captured molecules between a Smartseq3 cells and a Smartseq3xpress cell. Boxplots in **c**, **e**, **f** and **g** show the median, first and third quartiles as a box, and the whiskers show the most extreme data point within 1.5 lengths of the box.

Next, we realized that the Smart-seq3 TSO could mis-prime during RT to induce a strand invasion artifact^11,12^ (Extended Data Fig. 4a,b). We screened TSO sequences of various designs and higher RT temperatures (Supplementary Note 1) for their effect on mis-priming, accuracy in UMI-based counting, gene and RNA molecule sensitivity and general quality (Fig. 1l,m and Extended Data Fig. 4c,d). Although no TSO showed excellent performance across all metrics, new TSO sequences with improved performance compared to Smart-seq3 and other TSOs^12^ were identified. Next, we validated candidate TSOs in primary cells to confirm sensitivity (Extended Data Fig. 5a) and fine-tuned oligonucleotide primer concentrations for optimal performance (Extended Data Fig. 5b). Thus, Smart-seq3xpress with the improved TSO has substantially reduced strand invasion in both peripheral blood mononuclear cells (PBMCs) (Extended Data Fig. 5c) and HEK293FT cells (Extended Data Fig. 5d,e) while maintaining accurate RNA counting (Fig. 1l,m and Extended Data Fig. 4d).

**Extended Data Fig. 6:**
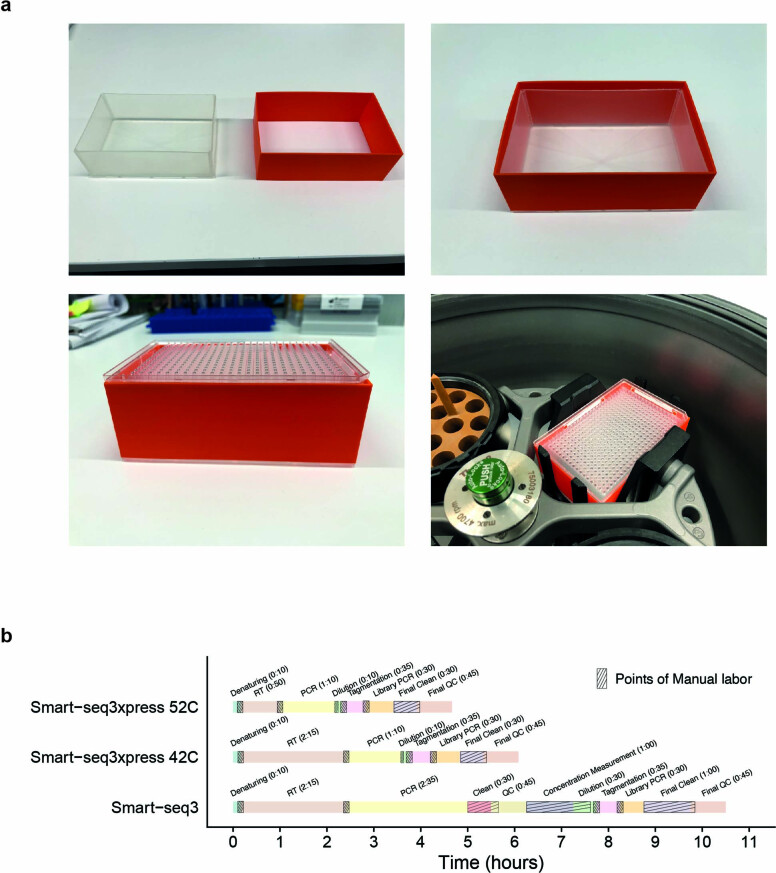
Experimental improvements to the Smart-seq3xpress workflow. (**a**) Introduction of a 3D-printed adapter to facilitate pooling of final libraries by centrifugation. Pictures showing reservoir and 3D printed frame/holder and assembly to facilitate pooling of 384 well plates quickly via gentle centrifugation. (**b**) For Smart-seq3 and Smart-seq3xpress (42 °C and 52 °C reverse transcription implementations), we show the workflow in the library preparation process with the associated timings. Any steps that require hands-on work are shaded with pattern.

Finally, we benchmarked both low-volume Smart-seq3 (1 µl, KAPA) and Smart-seq3xpress (12 PCR cycles, using either KAPA or SeqAmp with new and old TSO) against standard-volume Smart-seq3 (ref. ^2^). Notably, we observed an improved gene and molecule detection with SeqAmp-based Smart-seq3xpress (Fig. 1n and Extended Data Fig. 5f–h). Crucially, the material and resources needed to construct Smart-seq3xpress singe-cell libraries were ten-fold reduced, allowing researchers to substantially increase the cell numbers analyzed. Further streamlining and reduction of plastics consumables was achieved by collecting final libraries by centrifugation using a simple 3D-printed adapter (Extended Data Fig. 6a) and through contact-less combinatorial index dispensing or relying on tagmentation plates containing already dispensed desiccated index primers. Thus, the miniaturization and streamlining of Smart-seq3 was feasible at improved gene and molecular detection so that sequencing-ready Smart-seq3xpress libraries can be reached within a single workday (Extended Data Fig. 6b).

**Fig. 2:**
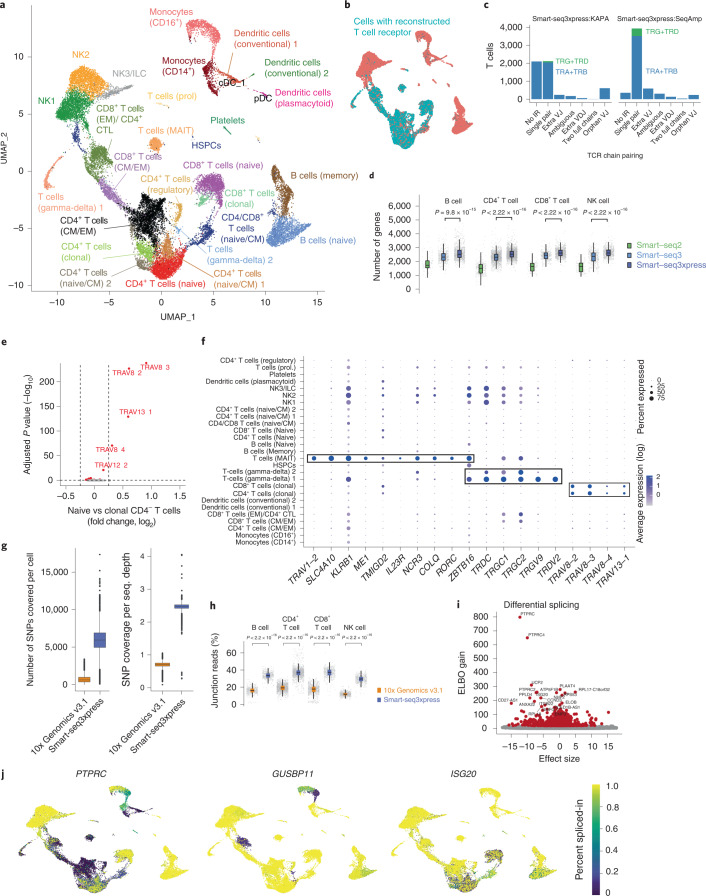
Application of Smart-seq3xpress to hPBMCs. **a**, Dimensional reduction (UMAP) of 26,260 hPBMC transcriptomes produced with Smartseq3xpress (KAPA, four donors; SeqAmp with improved TSO, three donors) colored and annotated by cell type. EM, effector memory; CM, central memory; NK, natural killer; ILC, innate lymphoid cell; HSPC, hematopoietic stem and progenitor cell; MAIT, mucosal-associated invariant T cell. **b**, Smartseq3xpress-based TCR reconstruction (TRaCeR) overlayed onto UMAP. **c**, QC of TCR reconstructions obtained with Scirpy, enumerating the number of T cells with certain types of TCR reconstructions. **d**, Benchmarking of Smart-seq2, Smart-seq3 and Smart-seq3xpress (SeqAmp, improved TSO) in primary hPBMCs. Each cell was downsampled to 100,000 reads, and the number of detected genes from exon-mapping reads is shown for representative cell types: B cells (*n* = 73, 366 and 859, respectively), CD4^+^ T cells (*n* = 261, 1,270 and 1,847, respectively), CD8^+^ T cells (*n* = 76, 272 and 913, respectively) and NK cells (*n* = 73, 352 and 601, respectively). *P* values indicate results of two-sided *t*-tests between the Smart-seq3 and Smart-seq3xpress. **e**, Differential gene expression analysis (Wilcoxon test, *P*_adj_ < 0.01) between naive CD4^−^ T cell cluster (*n* = 2,476) and clonal CD4^−^ T cell cluster (*n* = 682). Indicated are the top five TCR genes driving the clonal CD4^−^ T cell cluster separation. **f**, Dot plot showing expression of selected marker genes for MAIT, gamma-delta and clonal CD4^+^/CD8^+^ T cells in all annotated clusters, with size of the dot denoting the detection of a gene within the cells of the cluster and color scale denoting the average expression level. **g**, Analysis of captured transcribed genetic variation in donor-matched Smart-seq3xpress and 10x Genomics 3′ version 3.1 data. For each cell passing QC (*n* = 2,938 and 9,846, respectively), the number of SNPs with alternate allele coverage per cell are indicated (left) as well as the average SNP coverage normalized by the sequencing depth (right). **h**, Frequency of RNA-velocity-informative fully spliced reads in donor-matched Smart-seq3xpress and 10x Genomics 3′ version 3.1 data. For each cell in representative cell types—B cells (*n* = 404 and 642, respectively), CD4^+^ T cells (n = 1,320 and 1,317, respectively), CD8^+^ T cells (*n* = 417 and 1,181, respectively) and NK cells (*n* = 441 and 498, respectively)—we summarized the percentage of reads spanning exon–exon junctions, with nominal *P* values for a two-sided *t*-test. **i**, Differential splicing isoform analysis using BRIE2. Shown is a volcano plot of tested skipped-exon events color-coded by significant variation when testing for any cell type (ELBO gain >20). ELBO gain is a surrogate for the Bayes factor (that is, the likelihood ratio of two hypotheses). The *x* axis denotes the effect size on the distribution of PSI values in a cell type. **j**, Overlays of color-coded PSI values inferred for each sequenced cell (*n* = 26,260) in genes with significant cell type splicing variation (PTPRC, GUSBP11 and ISG20). The box plots (in **d**, **g** and **h**) show the median and first and third quartiles as a box, and the whiskers show the most extreme data points within 1.5 lengths of the box. HSPC, hematopoietic stem and progenitor cell.

**Extended Data Fig. 7:**
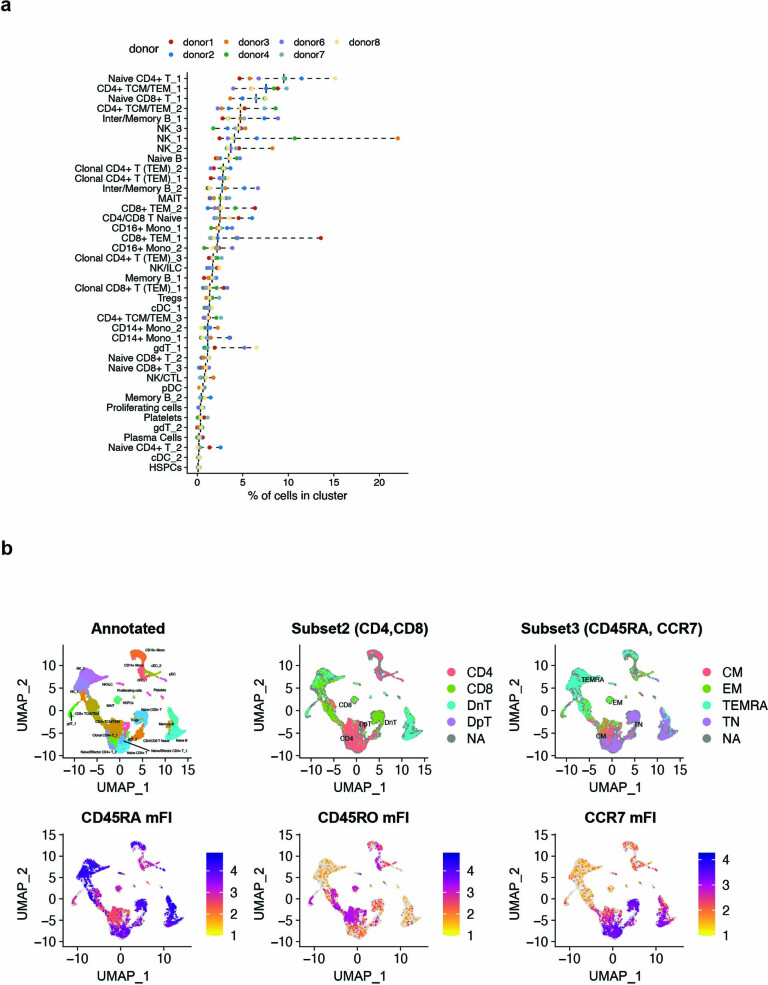
Analysis of PBMC samples using Smart-seq3xpress. (**a**) Distribution of cell type abundances as a percentage of all cells from each of the 7 donors. (**b**) FACS-based (index sorting) data overlayed onto UMAP embeddings. “Top Left” shows annotated UMAP based on gene expression. “Top middle”, shows classification of all sorted live cells based on their expression of CD4 and CD8. (DnT = double negative CD4- and CD8-, DpT = Double positive CD4 + and CD8 + , NA = cells without staining). “Top Left” all recorded, and index sorted live cells are categorized based on their expression of CD45RA and CCR7 overlayed over UMAP embeddings (CM = CD45RA- CCR7 + , EM = CD45RA- CCR7-, TEMRA = CD45RA + CCR7-, TN = CD45RA + CCR7 + , NA = cells without stainings). “Lower panels” show protein levels for antibody-staining against CD45RA, CD45RO and CCR7 overlayed over UMAP embeddings of sequenced transcriptomes as log-scaled mean fluorescence intensity (mFI).

**Extended Data Fig. 8:**
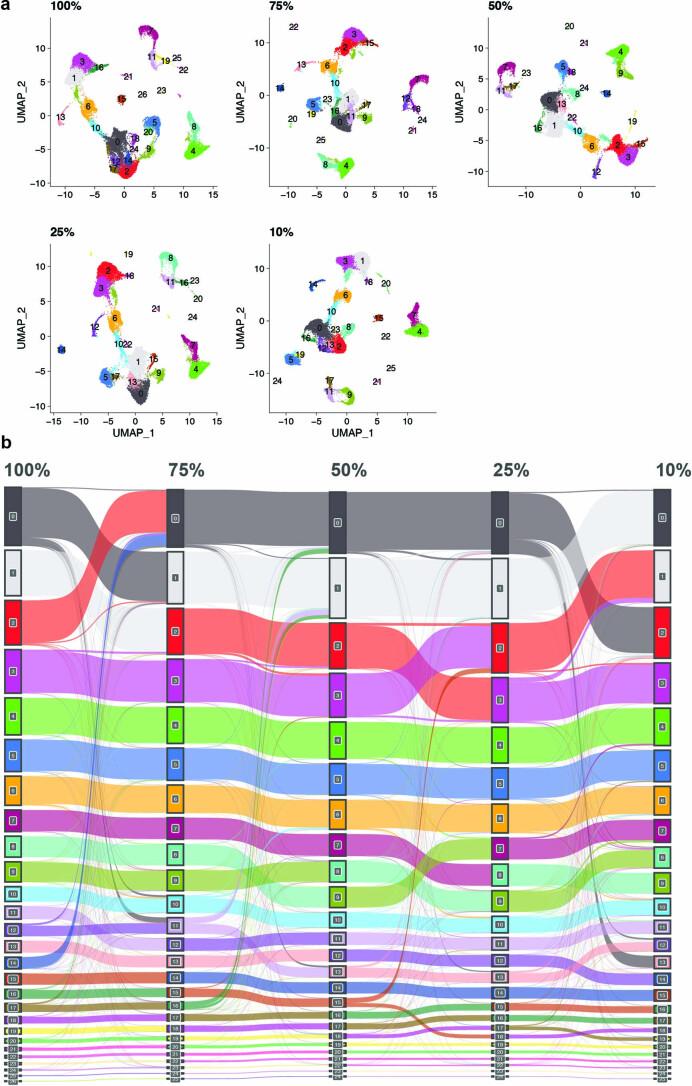
Downsampling of Smart-seq3xpress PBMC data. Sequencing data generated for 26,260 human PBMCs was downsampled from a median depth of 258,000 read pairs per cell to steps of 75% (~193,000 reads), 50% (~129,000 reads), 25% (~64,000 reads), 10% (~26,000) of reads while retaining the relative abundances of per-cell coverage. (**a**) At each of the downsampling depths, we repeated the Seurat workflow of normalization, clustering and dimensionality reduction using UMAP. (**b**) We tracked the assigned cluster identity of every cell in the dataset over the downsampling depths and visualized the flow of clustered cells by connecting cluster labels (nodes) with line widths scaled to the number of cells.

To showcase Smart-seq3xpress, we profiled 26,260 human peripheral blood mononuclear cells (hPBMCs) at an average depth of 258,000 read pairs per cell. Sequence data were demultiplexed, processed and quality controlled with zUMIs^13^, and, after stringent QC, Seurat^14^ was used for downstream analysis (Methods). The single-cell transcriptomes were visualized with uniform manifold approximation and projection (UMAP) and separated into 27 clusters (Fig. 2a) that were supported by all donors (Extended Data Fig. 7a) and protein staining from the index sorting (Extended Data Fig. 7b). Reconstruction of T cell receptor (TCR) sequences from the internal reads matched the identified T cell clusters (Fig. 2b), and the bulk of reconstructed TCR sequences corresponded to expected single-chain pairing (Fig. 2c). Consistently higher gene detection was observed across cell types for Smart-seq3xpress in comparison to Smart-seq2 (ref. ^15^) and Smart-seq3 (ref. ^2^) (Fig. 2d). A recent study^14^ showed that scRNA-seq alone was not capable of separating certain cell types and states in hPBMCs without additional single-cell protein measurements. In a dataset with 200,000 hPBMC transcriptomes generated with 10x Genomics, 228 protein markers (CITE-seq) were used to distinguish unconventional T cell populations (mucosal-associated invariant T (MAIT) cells), gamma-delta T cells and effector memory T cells. All these cell types separated by the Smart-seq3xpress transcriptome data alone (Fig. 2a), exemplified with marker gene expression for MAIT cells, gamma-delta T cells and a CD4^+^ T cell population characterized only by their clonal expression of specific TCRs (Fig. 2e,f). Notably, this highly granular de novo cell type and state characterization was obtained from only 26,260 Smart-seq3xpress transcriptomes, and similar granularity remained at lower sequence depths (Extended Data Fig. 8).

**Extended Data Fig. 9:**
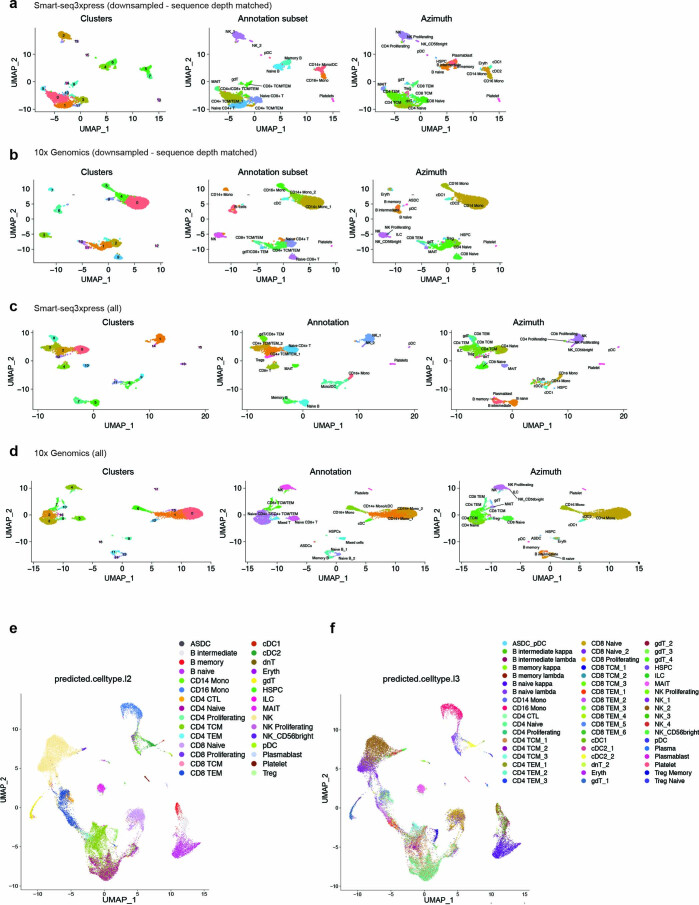
Direct comparison of Smart-seq3xpress and 10x Genomics 3’ v3.1 data. Human PBMCs from the same donor were processed and sequenced using both protocols. We sampled the same number of sequenced reads (approx. median 140,000 per cell) and cells (3,000) and applied an identical data analysis workflow using Seurat (see Methods). (**a**) Shown are the UMAP embeddings for Smartseq3xpress identifying the number of clusters (left), annotated cluster names (middle) and cluster identities by reference-based identification via Azimuth cell type predictions (level 2 annotations) (**b**) 10xv3.1 UMAP embeddings showing clusters found (left), annotated cluster names (middle) and reference based predicted annotations by Azimuth (prediction level 2). (**c**-**d**) Shows the full the donor matched data without downsampling or cell subsetting after QC for both Smartseq3xpress (n = 3187) and 10xv3.1 (n = 6483). (left) clusters detected, (middle) annotated cell types based on clusters, (right) reference-based azimuth cell type predictions (prediction level 2). (**e**-**f**) Reference-based annotation of all human PBMC generated in this study using Smartseq3xpress by Azimuth (Hao et al., 2020) using the human PBMC reference dataset available at https://azimuth.hubmapconsortium.org. (**e**) Smartseq3xpress UMAP is colored and annotated by Azimuth cell type annotation level 2. (**f**) Smartseq3xpress UMAP is colored and annotated by Azimuth cell type annotation level 3.

Next, we performed a direct comparison of Smart-seq3xpress to droplet-based 10x Genomics (10x Genomics 3′ version 3.1; Methods) on a matched hPBMC donor. Tabulating the coverage over transcribed single-nucleotide polymorphism (SNP) positions—which is, for example, important for studies of allele-specific expression and cancer—showed that more SNPs were covered in the full-length Smart-seq3xpress data (9.2 million versus 2.1 million positions, over all cells). On a per-cell level, the number of SNPs with alternate allele coverage was approximately nine-fold higher with Smart-seq3xpress and with a three-fold-higher coverage per sequenced read (Fig. 2g). Similarly, full-length coverage with Smart-seq3xpress resulted in significantly (2–5-fold) increased read support over exon–exon and exon–intron splice junctions, which form the basis for RNA velocity inference^16^ (Fig. 2h). To perform a direct comparison of cluster granularity, we sampled an equal number of mean sequenced reads and cells for both 10x Genomics and Smart-seq3xpress and performed independent cluster analyses (Extended Data Fig. 9). Although both methods had approximately similar granularity at this number of cells and read numbers, cell type prediction based on Azimuth was slightly more consistent with clustering in Smart-seq3xpress (adjusted Rand index (ARI) of 0.59 and 0.49, respectively).

**Extended Data Fig. 10:**
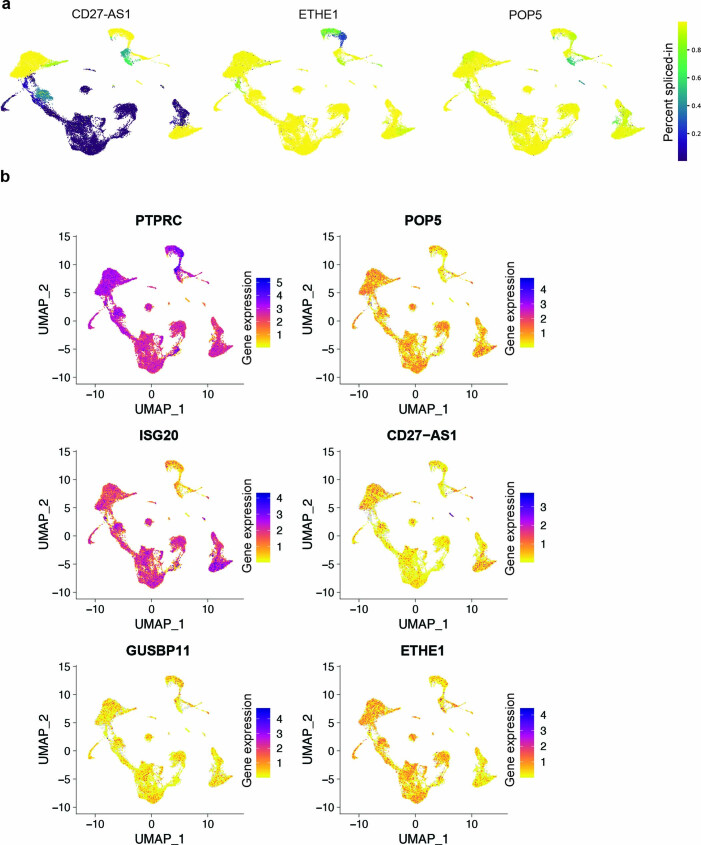
PBMC splicing analysis using Smart-seq3xpress. (**a**) Percent-spliced-in (PSI) for skipped exon events in the CD27-AS1, ETHE1 and POP5 genes are overlayed as color scale on the UMAP embedding of sequenced PBMC cells (n = 26,260). (**b**) For genes with significant cell-type specific splicing patterns (shown in Figures. 3j, Supplementary Fig. 18a), scaled expression levels (log-normalized read counts) are annotated as colors.

To study alternative splicing across the hPBMC Smart-seq3xpress data, we used BRIE2 (ref. ^17^) to identify 968 skipped exons with significant variation in inclusion levels across cell types. (Fig. 2i). Overlaying cell-level percent spliced-in (PSI) estimates for alternative exons onto the UMAP revealed distinct splicing patterns across cell types. These included switching, graded changes, as well as heterogeneous cell types with variable inclusion levels (Fig. 2j), and, notably, they were independent of their gene-level expression levels between cell types (Extended Data Fig. 10). Thus, Smart-seq3xpress can generate unprecedented high-quality, full-transcript-coverage scRNA-seq data that enable scalable analyses of alternative splicing across complex human samples.

Large-scale efforts to enumerate cell types and states across human tissues and in model organisms are dominated by scRNA-seq methods that count RNAs by sequencing their ends. With the development of Smart-seq3xpress, we demonstrate a scalable solution for full-transcript-coverage scRNA-seq. Not only has Smart-seq3xpress overcome the main limitations of plate-based assays in terms of resources and material needed per cell, the data obtained also challenge the existing notions of the cell numbers required for efficient and finely resolved clustering of cells. Therefore, high-sensitivity scRNA-seq with isoform-specific and allele-specific resolution can, for the first time, be performed with Smart-seq3xpress at a scale suitable for large-scale cell atlas building.

## Methods

### Cell sources, culturing and sorting

HEK293FT cells (Invitrogen) were grown in DMEM medium (4.5 g L^−1^ of glucose and 6 mM L-glutamine, Gibco), supplemented with 10% FBS (Sigma-Aldrich), 0.1 mM MEM non-essential amino acids (Gibco), 1 mM sodium pyruvate (Gibco) and 100 µg ml^−1^ of pencillin–streptomycin (Gibco) at 37 °C. For sort of single cells, cells were harvested by incubation with TrypLE Express (Gibco). K562 (ATCC) cells were grown in RPMI medium, supplemented 10% FBS (Sigma-Aldrich) and 1% pencillin–streptomycin (Gibco). Frozen aliquots of 10 million hPBMCs from healthy individuals were purchased from Lonza, requiring healthy donors only. Written informed consent was obtained at sampling point from all donors by Lonza, and our analyses of hPBMCs were approved by the Regional Ethical Review Board in Stockholm, Sweden (2020-05070). hPBMCs were gently thawed and stained with PE mouse anti-human CCR7 (2-L1-A,1:100), PE-Cy7 mouse anti-human CD4 (SK3,1:250), FITC mouse anti-human CD45RA (HI100, 1:100), PerCP-Cy5.5/BB700 mouse anti-human CD8 (RPA-T8, 1:250) and PE-Cy5 mouse anti-human CD45RO (UCHL1, 1:250) (BD Biosciences) before sorting. For all cell types, dead cells were gated out after staining with propidium iodide (Thermo Fisher Scientific). Live, single cells were sorted into 384-well plates containing lysis buffer using a BD FACSMelody (BD FACSChorus version 1.3 software) equipped with a 100-µm nozzle and plate cooling with index sorting on (BD Biosciences). After sorting, each plate was immediately spun down and stored at −80 °C.

### Smart-seq3 library preparation

Full-volume Smart-seq3 library preparations were performed as previously described^2^. PCR was carried out using 20 cycles of amplification.

### Low-volume Smart-seq3 and Smart-seq3xpress library preparation

For experiments including overlays, including Vapor-Lock (Qiagen), silicon oils (Sigma-Aldrich) and tri-/tetradecane (Sigma-Aldrich), 3 µl of designated overlay was added to each well and stored at room temperature until use. Lysis buffer of various volumes (0.1–3 µL) was dispensed using either Formulatrix Mantis or Dispendix I.Dot One liquid dispenser to each well, all containing 0.1% Triton X-100, 5% PEG8000 adjusted to RT volume, 0.5 µM oligo(dT) adjusted to RT volume, 0.5 mM dNTPs each adjusted to RT volume and 0.5 U RNase Inhibitor (Takara, 40 U µl^−1^). After dispensing, lysis plates were briefly centrifuged down to ensure that lysis is properly collected and stored under the overlay. Stored plates of sorted cells were denatured at 72 °C for 10 minutes, followed by the addition of indicated volumes of RT mix; common for all is that the reagent concentrations are stable: 25 mM Tris-HCl ~pH 8.3 (Sigma-Aldrich), 30 mM NaCl (Ambion), 0.5 mM GTP (Thermo Fisher Scientific), 2.5 mM MgCl_2_ (Ambion), 8 mM DTT (Thermo Fisher Scientific), 0.25 U µl^−1^ of RNase Inhibitor (Takara), 2 µM TSO (5′-Biotin-AGAGACAGATTGCGCAATGNNNNNNNNrGrGrG-3′; IDT) and 2 U µl^−1^ of Maxima H Minus reverse transcriptase (Thermo Fisher Scientific). After RT mix dispensing, the plate was spun down to ensure merge of RT and lysis reactions. RT was performed at 42 °C for 90 minutes, followed by ten cycles of 50 °C for 2 minutes and 42 °C for 2 minutes. Indicated volumes of PCR master mix were dispensed, all containing constant reaction concentrations of 1× KAPA HiFi PCR buffer (Roche), 0.3 mM dNTPs each (Roche), 0.5 mM MgCl_2_ (Ambion), 0.5 µM Smart-seq3 forward primer (5′-TCGTCGGCAGCGTCAGATGTGTATAAGAGACAGATTGCGCAATG-3′; IDT), 0.1 µM Smart-seq3 reverse primer (5′-ACGAGCATCAGCAGCATACGA-3′; IDT) and 0.02 U µl^−1^ of KAPA HiFi DNA polymerase (Roche). After dispensing, the plate was quickly spun down before being incubated in PCR as follows: 3 minutes at 98 °C for initial denaturation, 10–24 cycles of 20 seconds at 98 °C, 30 seconds at 65 °C and 2–6 min at 72 °C. Final elongation was performed for 5 minutes at 72 °C. For conditions after cDNA pre-amplification clean-up: 100 nl of water, ExoSAP-IT express (Thermo Fisher Scientific) or 0.5 U ExoI (NEB) + 0.05 FastAP (Thermo Fisher Scientific) was dispensed per well and incubated at 37 °C for 15 minutes, followed by inactivation at 85 °C for 5 minutes.

For Smartseq3xpress with SeqAmp (Takara), lysis and RT was carried out with 0.125 µM or 0.5 µM oligodT30VN and 0.75 µM or 2 µM TSO unless otherwise indicated as described above. Original Smartseq3 TSO (5′-Biotin-AGAGACAGATTGCGCAATGNNNNNNNNrGrGrG-3′; IDT). Improved TSO (5′-Biotin-AGAGACAGATTGCGCAATGNNNNNNNNWWrGrGrG-3′; IDT). PCR mastermix was dispensed at 0.6 µl per cell containing 1× SeqAmp PCR buffer, 0.025 U µl^−1^ of SeqAmp polymerase and 0.5 µM/1 µM Smartseq3 forward and reverse primer. After dispensing PCR mastermix, the plate was quickly spun down before being incubated as follows: 1 minute at 95 °C for initial denaturation, 6–18 cycles of 10 seconds at 98 °C, 30 seconds at 65 °C and 2–6 minutes at 68 °C. Final elongation was performed for 10 minutes at 72 °C. For Smartseq3xpress with NEBNext Ultra II Q5 Master Mix (NEB), PCR mastermix consisted of 1× NEBNext Ultra II Q5 Master Mix and 0.5 µM/1 µM Smartseq3 forward and reverse primer and PCR was performed at 30 seconds at 98 °C for initial denaturation, 12 cycles of 10 seconds at 98 °C, 30 seconds at 65 °C and 6 minutes at 72 °C. Final elongation was performed for 5 minutes at 72 °C. For Smartseq3xpress with NEBNext Q5 Hot Start HiFi PCR Master Mix (NEB), PCR mastermix consisted of 1× NEBNext Q5 Hot Start HiFi PCR Master Mix and 0.5 µM/1 µM Smartseq3 forward and reverse primer and PCR was performed for 30 seconds at 98 °C for initial denaturation, 12 cycles of 10 seconds at 98 °C, 30 seconds at 65 °C and 1 minute at 65 °C. Final elongation was performed for 5 minutes at 65 °C. For Smartseq3xpress with Platinum SuperFi II DNA polymerase (Invitrogen), PCR mastermix consisted of 1× SuperFi II Master Mix, 0.2 µM dNTPs and 0.5 µM/1 µM Smartseq3 forward and reverse primer and PCR was performed for 30 seconds at 98 °C for initial denaturation, 12 cycles of 10 seconds at 98 °C, 30 seconds at 60 °C and 6 minutes at 72 °C. Final elongation was performed for 5 minutes at 72 °C. For Smartseq3xpress with Platinum II Taq Hot Start DNA polymerase (Invitrogen), PCR mastermix consisted of 1× Platinum II Taq Master Mix, 0.2 µM dNTPs and 0.5 µM/1 µM Smartseq3 forward and reverse primer and PCR was performed for 2 minutes at 94 °C for initial denaturation, 12 cycles of 15 seconds at 94 °C, 30 seconds at 60 °C and 6 minutes at 68 °C. Final elongation was performed for 5 minutes at 68 °C.

A full and comprehensive protocol of Smart-seq3xpress has been deposited on protocols.io^18^.

### After pre-amplification workflow

For regular Smart-seq3, pre-amplified cDNA libraries were purified with homemade 22% PEG beads at a ratio of 1:0.6. Library sizes were observed using Agilent Bioanalyzer High Sensitivity Chip, followed by concentration quantification using QuantiFlour dsDNA assay (Promega). cDNA was subsequently diluted to 100 pg µl^−1^ unless otherwise specified.

For low volume, pre-amplified cDNA libraries were diluted by the addition of 9 µl of water to each well, if not indicated otherwise, followed by a quick centrifugation. Library sizes were checked on an Agilent Bioanalyzer, using the high-sensitivity DNA chip; meanwhile, concentrations were quantified using QuantiFlour dsDNA assay (Promega). cDNA was normalized to 100 pg µl^−1^ if nothing else was specified.

For Smart-seq3xpress, pre-amplified cDNA libraries were diluted with the addition of 9 µl of water unless stated otherwise, before transferring 1 µl of diluted cDNA from each well into tagmentation.

### Sequence library preparation for Smart-seq3xpress

Tagmentation was performed in 2 µl consisting of 1 µl of either diluted or normalized pre-amplified cDNA and 1 µl of 1× tagmentation buffer (10 mM Tris pH 7.5, 5 mM MgCl_2_, 5% DMF), 0.025–0.5 µl of ATM (Illumina XT DNA sample preparation kit) or 0.0002–0.01 µl of tagmentation DNA enzyme 1 (TDE1;Illumina DNA sample preparation kit)). In the event of in-house Tn5, 1× tagmentation buffer used consisted of 10 mM TAPS-NaOH pH 8.4, 5 mM MgCl_2_ and 8% PEG8000 and indicated amounts of 0.0005–0.01 µM in-house Tn5 enzyme. Samples were incubated at 55 °C for 10 minutes, followed by the addition of 0.5 µl of 0.2% SDS to each well. After addition of 1.5 µl/3.5 µl of custom Nextera index primers (0.5 μM) carrying 10-bp dual indexes, library amplification was started by the addition of 2/4 µl of PCR mix (1× Phusion Buffer (Thermo Fisher Scientific), 0.01 U µl^−1^ of Phusion DNA polymerase (Thermo Fisher Scientific), 0.2 mM dNTP each) and incubated for 3 minutes at 72 °C; 30 seconds at 95 °C; 12–14 cycles of (10 seconds at 95 °C; 30 seconds at 55 °C; 30–60 seconds at 72 °C); and 5 minutes at 72 °C in a thermal cycler. Samples were pooled by spinning out each plate gently in a 300-ml robotic reservoir (Nalgene) fitted with a custom 3D-printed scaffold by pulsing to ~200*g*. The pooled library was purified with homemade 22% PEG beads at a ratio of 1:0.7.

### 10x Genomics library preparation

After thawing the PBMC sample, we stained dead cells with propidium iodide (Thermo Fisher Scientific) and sorted 200,000 live cells into a 5-ml tube. After centrifugation, the cell suspension was resuspended and concentration of cells was determined using a Countess automated cell counter (Thermo Fisher Scientific). We loaded ~13,000 cells for a target cell recovery of ~8,000 cells and prepared libraries according to the 10x Genomics version 3.1 user guide. For both pre-amplification and post-fragmentation PCR, we applied 12 cycles of PCR.

### Sequencing

Smartseq3 and Smartseq3xpress libraries were sequenced on a Illumina NextSeq 500 (Illumina NextSeq Control Software 2.2.0) or MGI DNBSEQ G400RS platform (version 1.1.0.108 software). For NextSeq runs with Smart-seq3, denatured libraries were loaded on HighOutput version 2.5 cartridges at 2.1–2.3 pM. For G400RS runs, libraries were created using phosphorylated index primers or subjected to five cycles of adapter conversion PCR using the MGIEasy Universal Library Conversion Kit (MGI) and subsequently circularized from 1 pmol of dsDNA according to the manufacturer’s protocol. Next, 60 fmol of circular ssDNA library pools were used for DNA nanoball (DNB) making using a custom rolling-circle amplification primer (5′-TCGCCGTATCATTCAAGCAGAAGACG-3′). DNBs were loaded on FCL flow cells (MGI) and sequenced using SE100, PE100 or PE150 cartridges using custom sequencing primers (Read 1: 5′-TCGTCGGCAGCGTCAGATGTGTATAAGAGACAG-3′; MDA: 5′-CGTATGCCGTCTTCTGCTTGAATGATACGGCGAC-3′, Read 2: 5′-GTCTCGTGGGCTCGGAGATGTGTATAAGAGACAG-3′; i7 index: 5′-CCGTATCATTCAAGCAGAAGACGGCATACGAGAT-3′; i5 index: 5′-CTGTCTCTTATACACATCTGACGCTGCCGACGA-3′). 10x Genomics version 3.1 libraries were sequenced on a NextSeq 500 according to manufacturer specifications (1.8 pM loading concentration; HighOutput version 2.5 150-cycle kits, 28–8–92 cycles for read1, index1 and read2).

### Primary data processing

zUMIs^13^ version 2.8.2 or newer was used to process raw FASTQ files. Reads were filtered for low-quality barcodes and UMIs (4 bases < phred 20, 3 bases < phred 20, respectively) and UMI-containing reads parsed by detection of the pattern (ATTGCGCAATG) while allowing up to two mismatches. Reads were mapped to the human genome (hg38) using STAR version 2.7.3, and error-corrected UMI counts were calculated from Ensembl gene annotations (GRCh38.95). zUMIs was also used to downsample cells to equal raw sequencing depth to facilitate method benchmarking.

### Analysis of Smartseq3xpress hPBMC data

Cells were filtered for low-quality libraries, requiring (1) more than 50% of read pairs mapped to exons+introns, (2) more than 20,000 read pairs sequenced, (3) more than 500 genes (exon+intron quantification) detected per cell and (4) less than 15% of read pairs mapped to mitochondrial genes. Furthermore, a gene was required to be expressed in at least ten cells. Analysis was done using Seurat v4.0.1 (ref. ^14^). Data were normalized (‘LogNormalize’), scaled to 10,000 and total number of counts, and mitochondrial fraction was regressed out. Using the Seurat integration function, the donor effect from the seven different donors in the dataset was removed. The top 10,000 variable genes were considered and 35 principal components for shared nearest neighbor (SNN) neighborhood construction and UMAP dimensionality reduction. Cell clusters were produced using Louvain algorithm at a resolution of 0.8. Cell types were identified by using the R package Presto (Wilcoxon & AUC, version 1.0.0). For the Azimuth predictions, a QC-filtered count matrix was uploaded to the Azimuth web-based application and processed according to the Azimuth app. For the direct donor comparison with 10x Genomics version 3.1 data, read counts from only donor 7 were downsampled to similar median sequencing depth as the comparable 10x dataset and quality filtered as follows: at least 10,000 read pairs, more than 50% of read pairs mapped to exons+introns and less than 15% read pairs mapped to mitochondrial genes. A gene was required to be expressed in at least ten cells. Data were subset to 3,000 cells for analysis in Seurat. Data were LogNormalized, scaled to 10,000 and mitochondrial genes regressed out. Default Seurat settings were used for neighborhood construction and dimensionality reduction. Cell clusters were assigned using the Louvain algorithm at a resolution of 0.8. Cell type identification was performed as above.

### Analysis of 10x Genomics version 3.1 donor 7 hPBMC data

Raw sequencing data in FASTQ format were processed using zUMIs version 2.9.3 with automatic barcode detection based on the 10x Genomics version 3.1 allow-list. After completion, we exported full count tables including empty droplets and assigned ambient RNA droplets and real cells using the CellBender (version 0.2.0) remove-background function^19^. To filter for doublets, the CellBender output.h5 file was used with Solo^20^ (version 0.6). Additional doublets were discarded by manually inspecting the distribution of total UMI counts per droplet and discarding those greater than 45,000. For downstream analysis in Seurat, a low-quality filter was applied based on requiring at least 10,000 read pairs and less than 10% read pairs mapped to mitochondrial genes. A gene should be expressed in at least ten cells to be included. A subset of 3,000 cells out of 6,483 passing QC was used for direct comparison to Smart-seq3xpress. Seurat was run at default settings using SCTransform, and cell clusters were assigned at resolution 0.8 using the Louvain algorithm. Cell types were identified using Presto (Wilcoxon & AUC) together with reference-based approach performed by the Azimuth app.

### TCR reconstruction

TCR sequences were reconstructed using TraCeR version 0.6.0 (ref. ^21^) run in the teichlab/tracer Docker environment and using the --loci A B G D --species Hsap flags. Scirpy^22^ (version 0.8.0) was used to summarize and QC the output from TraCer.

### Molecular spike data processing and analysis

Molecular spike data were extracted from aligned zUMIs BAM files and analyzed using the UMIcountR package^10^ (https://github.com/cziegenhain/UMIcountR, version 0.1.1). After loading the data using the ‘extract_spike_dat’ function, overrepresented spikes were discarded with a read cutoff of 25 and higher. We next used molecular spike observations across all cells and conditions with at least five reads per molecule to sample 26 ground truth mean expression levels from 1 to 316 molecules per cell using the ‘subsample_recompute’ function. We then plotted the mean counting difference shaded by the standard deviation.

### Identification of TSO strand invasion artifact

To identify UMI reads with the TSO mis-primed artifact, we loaded sequencing reads into R using Rsamtools (version 2.6.0). In the case of paired-end sequencing, only first-in-pair reads were selected using the appropriate SAM flags. The strand orientation of the mapped reads was also determined from SAM flags. Then, we extracted a 20-bp window of genome sequence upstream of the read start position on the positive strand (+stranded mappings) or downstream of the read start position+read length on the negative strand (−stranded mappings) using the BSgenome package (version 1.62.0, human hg38). Afterwards, we checked for presence of the UMI sequence (with or without addition of GGG overhang) in the genomic window using R’s fuzzy string matching function (allowing 0, 1 or 2 mismatches). This identification procedure of artifactual UMI reads was also implemented in Python3 to process aligned BAM files and remove all artifactual reads/read pairs (available on GitHub: https://github.com/cziegenhain/pyTSOfilter).

### Isoform-based analysis

For analysis of skipped-exon (SE) isoform differences, we retrieved annotations from GenCode (Human v39) and produced the SE annotation in GFF file format using BRIEkit-event (version 0.2.2). We filtered SE events using BRIEkit-event-filter with the following criteria: (1) retain SE events on autosomes and X/Y chromosomes; (2) SE events not overlapped by any other AS-exon; (3) surrounding introns are no shorter than a fixed length (100 bp); (4) presence of specific splice sites (that is, surrounded by AG-GT); and (5) SE events have a minimum distance (10 bp) from transcription start site or transcription termination site. Next, we summarized the coverage over the filtered SE events for each cell using the brie-count command from BRIE2 (version 2.0.6) using per-cell demultiplexed, aligned and TSO-artifact-filtered (see above) BAM files as input. The resulting count files in h5ad format were used as input for the Bayesian regression-based inference of PSI values and variable splicing detection over cell types. We applied the aggregated imputation mode introduced by BRIE2 to fit the gene-wise prior distribution through aggregation of data over all cells for each gene. Default settings for Monte Carlo EM were applied. Genes were filtered by requiring at least 50 counts, ten unique counts and at least 30 cells with unique counts. The minimum required minor isoform frequency was 0.001 (default settings). For variable splicing detection, we annotated each cell with a binarized dummy factor of cell type identity (Seurat clustering; Louvain resolution 2.0), removing the most common cell type to avoid collinearity of the design matrix. We loaded the resulting h5ad file into scanpy^23^ (version 1.8.2) for visualization of PSI values. For selection of SE events with significant cell type difference, we selected the highest evidence lower bound (ELBO) value per SE for each of the cell type LRT indices. Gene model plots to visualize significant cell-type-variable SE events were generated using the Gviz (version 1.38.1L, https://link.springer.com/protocol/10.1007%2F978-1-4939-3578-9_16) and rtracklayer (version 1.54.0, https://academic.oup.com/bioinformatics/article/25/14/1841/225816) R packages.

### SNP and junction coverage analysis

Coverage over transcribed SNPs was analyzed per cell using the cellsnp-lite^24^ package (version 1.0.0) over the most common human polymorphisms (1000 Genomes Project minor allele frequency >0.005, 36 million positions). We applied default settings of minimum aggregated count over cells 20 and minimum MAPQ for read filtering of 20 (essentially discarding multimapping reads due to the mapping quality encoding of the STAR aligner). Coverage on RNA velocity informative positions was tabulated from zUMIs output BAM files. Fully spliced exon–exon reads were identified by the presence of splicing in their CIGAR value and exclusive assignment to exon regions, whereas nascent (that is, unspliced or partially spliced) exon–intron spanning reads were identified by the overlap with both exonic and intronic regions of the same gene.

### Reporting summary

Further information on research design is available in the Nature Research Reporting Summary linked to this article.

## Online content

Any methods, additional references, Nature Research reporting summaries, source data, extended data, supplementary information, acknowledgements, peer review information; details of author contributions and competing interests; and statements of data and code availability are available at 10.1038/s41587-022-01311-4.

## Supplementary information

Supplementary Information Supplementary Note 1

Reporting Summary

## Data Availability

Sequencing data have been deposited at ArrayExpress under the following accession numbers: E-MTAB-11488, E-MTAB-11452 and E-MTAB-11467.

## Code availability

Processing of Smart-seq3xpress data was implemented in zUMIs (https://github.com/sdparekh/zUMIs). Code to filter reads with the TSO strand invasion artifact was implemented in a stand-alone script pyTSOfilter (https://github.com/cziegenhain/pyTSOfilter).
