# Supplementary material for: Scalable single-cell RNA sequencing from full transcripts with Smart-seq3xpress
Source: Nat Biotechnol. 2022 May 30;40(10):1452–7. doi: 10.1038/s41587-022-01311-4 (PMC9546772; doi:10.1038/s41587-022-01311-4)
Supplement: Supplementary file 1 — Supplementary Note 1 [file 41587_2022_1311_MOESM1_ESM.pdf]

nature portfolio

https://doi.org/10.1038/s41587-022-01311-4

Supplementary information

Scalable single-cell RNA sequencing from full transcripts with Smart-seq3xpress

In the format provided by the authors and unedited

Supplementary Note. Further details on method design, decisions, and considerations.

Overall design. Smart-seq3xpress is designed to be a much cheaper and more streamlined version of Smart-seq3 facilitating easier production of larger experiments, without sacrificing features and quality. Time-consuming steps from Smart-seq3, e.g. cDNA library QC, excessive preamplification PCR, that result in the need to measure and normalize library concentrations, have been removed to save time, money, and resources, in such a manner that sequencing ready libraries can performed within a day. In this supplementary note we provide (1) general guidelines to the method, (2) describe decisions made during optimization and (3) considerations to keep in mind when using the method. This method is designed to be used with non-contact liquid handlers capable of dispensing 100 nL as a lower limit. We have tested the method and are currently running it on both Formulatrix Mantis and Dispendix I-Dot liquid handlers, however other solutions may work similarly well (eg. BioNex Nanodrop II).

Overlays. Most of the overlays used and tested in the current study are hydrophobic silicone-based liquids, inert to the reaction conditions and procedure. The concept of adding an overlay allow us to miniaturize by creating a barrier encapsulating the reaction mixtures. Furthermore, we postulated that it would also be able to act as a “landing cushion” for sorted cells, hence we scaled the amount of overlay to be like the original lysis volume (3uL) to accommodate greater FACS sorting success-rate.

We primarily use the commercial product VaporLock from Qiagen for the experiments here, since it was the first overlay evaluated. The hydrophobicity and low viscosity are beneficial but come with unusual side-effects, such as reduced adhesion of plate seals after general storage and storage in -80 °C. Thus, spilling on top of the plate is to be avoided but plate handling is not more problematic in general. Since then, we tested many different potential overlays to find alternatives and/or other certain chemical properties. For example, for shipping of plates, higher viscosity silicone oils (100 cSt and above) are preferred to lessen the chance of the overlay leaking out of wells during transportation. Similarly, we tested a wide variety of hydrocarbon solutions in the pursuit of different solidifying temperatures. In terms of pricing, Silicon Oil from Sigma (5-100 cSt) was the cheapest alternative.

Importantly, the hydrophobic overlays get added first to the 384 well plates for several reasons. First, 384 well plates containing just overlay can be prepared in bulk and stored, awaiting lysis buffer when needed. Second, dispensing of overlay is best done using traditional pipetting, either manual (8-16 channel pipette), semi-manual (Integra Viaflow or similar) or automatic (Agilent Bravo, Tecan Fluent etc.). **CAUTION:** Using these low viscosity hydrophobic liquids in non-contact liquid handlers can potentially severely damage them unless explicitly endorsed by the manufacturer. The overlay solutions are extremely hydrophobic and low viscosity which mean they can “creep” to sensitive parts (e.g., air ribbons).

Keep all stored plates (empty plates, frozen lysis plates, sorted plates) with overlay in upright orientation, as the overlay will run out of the well. Leftover or spilt overlay on the top of the plates will inhibit proper sealing of plates seals and adhesive film. Variations in the amount of overlay are however not critical as long as reaction volumes are encapsulated fully. Similarly

having “leaky” seals is also not a general issue, since the reactions are encapsulated below the overlay which inhibits evaporation. Reusable silicone sealing mats (such as Axygen AxyMats) can be substituted for adhesive seals.

The reaction volumes in Smart-seq3xpress are greatly reduced. To ensure that each dispensed volume meets the preexisting volume under the overlay, it is advised to follow up each dispense with a quick pulse or spin to ~1000g.

Cell sorting: To date we have tested successfully Smartseq3xpress when sorting cells into overlays on several popular FACS machines and cell printer, including BD FACSMelody, BD Fusion, BD Influx, Sony SH800S, Cellenion CellenOne, Cytena F.SIGHT2.0. For cell sorting, please consider the following. The lysis volume is now much less (300 nL). This means that the sorting / cell dispensing volume matters. Usually, most FACS sorters dispense the single cell in a droplet around ~5nL (for 100um nozzle). Some cell dispensing instruments dispense single cells in higher volumes 0.5uL-1uL or more and are therefore not suitable for this method. Similarly, consider your buffer of choice for sorting, as PBS alone can in large amounts interfere with downstream reactions. The dispensed cell volume now comprises of a relatively larger ratio of the single cell droplet to lysis and downstream, reaction mixes; hence one should be careful with amount of certain common additives like EDTA, FBS and BSA. In general, we recommend washing and prepare cells in any buffer of choice (including FBS/BSA/EDTA), but sorting in PBS alone, or as recommended for 10x Genomics, a solution of PBS + 0.04% BSA. We recommend use of PBS not containing Ca2+ or Mg2+ supplement.

Lysis: We tested a wide range of lysis volume increments (3 - 0.3uL) for downscaling reaction volumes. We have successfully also made high quality libraries at 0.2 and 0.1uL lysis volumes, which resulted in total reaction end volumes of 0.8 and 0.5uL respectively (data not shown). We found that scaling whilst keeping the reaction concentration and volume ratios between lysis, RT and PCR the same worked best and yielded overall, most stable results. We chose to settle on using the 1/10 scale (0.3 uL lysis) for three main reasons. First, the monetary return in saved reagents through scaling further diminishes (since the miniaturized reagents make up smaller parts compared to plastics etc that are constant). Second, the deposited cell volume becomes a larger factor that will influence the overall reaction efficiency, and 0.3ul lysis buffer is more robust to this variation. Third, choosing to not push the dispense amount to the limit of what is technically possible on nanodispensing instruments results in overall smaller variability when making libraries.

To protect from negative effects (reduced library complexity) due to PCR side products caused by PCR polymerase change or TSO redesigns (discussed later), especially in low RNA containing cells, we found that lowering oligo-dT primer amount from 0.5 uM to <= 0.125 uM could help alleviate these symptoms.

Reverse transcription & template switching oligo: For this crucial part of the method, we tested an array (>500) of RT conditions in more than 30,000 HEK293FT cells. We and others[1,2] had noticed that the Smart-seq3 TSO tended to mis-prime during RT, also termed strand invasion, causing generation of a small portion of artifactual UMI observations, where the sequence of the UMI matched the genomic sequence upstream of the mapped cDNA read start site (20 bp window). One solution to remedy this issue is to introduce a spacer sequence

in between UMI and rGrGrG (Ref[2]). Whilst this is true, it does however introduce another severe flaw. Inherently in the design of Smart-seq3 and Smart-seq3xpress, PCR primers (in particular the forward primer targeting the 5′ end) need to be able to outcompete the TSO in PCR to count UMIs correctly and avoid UMI-inflation because of incorporation of new TSO-UMI sequences during PCR[3,4]. Adding the proposed extra 5-6nt of spacer in TSO makes it impossible with the current design to account for this miscounting (Figure 2f,g and Supplementary Figure 9), because increased binding energy leads to a diminished competitive advantage of forward primer. To overcome this, we evaluated close to 100 different TSO designs with variations of the spacer solution to find the optimal compromise of limiting strand invasion without sacrificing the ability to count correctly, or on the overall complexity in terms of genes captured. To date the best alternative TSO incorporated two "W" bases (either A or T) as a spacer, that greatly diminished the strand invasion issue and still maintaining high counting accuracy and complex libraries to our standards.

Strand invasion also seemed to be correlated to excess TSO, and we found that reducing the TSO amount could also decrease the issue. Too low TSO concentrations, however, will affect the overall library quality, but we found that a reduction from 2 uM to 0.75 uM showed no difference in complexity, and as such became the new TSO concentration to use. An added benefit also being less occurrence of primer dimers, making the overall reaction more efficient.

There are other ways to combat strand invasion that we also explored. One solution is to raise the RT temperature, making it less favorable for the TSO to mis-prime. Even though we evaluated many conditions, with various RT temperatures and times, we were not able to retain the library complexity we strived for whilst especially at 52 °C strand invasion was nonexistent. Thus, we decided to keep the original RT temperature 42 °C with the new TSO design that overall yielded the best results. An overview of all the reaction conditions included and tested can be found in Supplementary Table 1.

PCR: One of the changes for Smartseq3xpress is the switch from KAPA to SeqAmp DNA polymerase. The need for this change is rooted in the overall design of the method having both UMI-containing 5′ reads and internal full transcript coverage reads. Ideally, we observe both types in sequencing and the ratio between the two is tunable to the experimental need, governed by the amount of tagmentation is performed on the library. Since the 5′-end of the preamplified library prior to tagmentation carries the PCR priming site for the Illumina i5 index, low amounts of tagmentation will favor UMI reads. Meanwhile the more cut sites are introduced with increasing amount of tagmentation, the more internal reads are present in the final library. Thus, high amount of tagmentation favors internal reads. Modulating this ratio or balance is inherent to the method and depends on each researcher's requirements for the experiment at hand. Smartseq3xpress should retain this possibility of modulation while still being cheap. However, we noticed that KAPA-amplified libraries were not modulated without spending excessive amounts on large amounts of Tn5 enzyme. This is the likely outcome of KAPA buffer being high in salt, which is known to cause template blocking for Tn5. This is of course only an issue for Smartseq3xpress since we only rely on diluting out the preamplified library before tagmentation as opposed to the established bead clean-up (see relevant section below). Hence, we decided to test various other commercially available DNA polymerases to find a suitable substitute. All the tested polymerases yielded complex

libraries in HEK293FT cells, although SeqAmp stood out as being slightly better in terms of the UMI ratio modulation and complexity (Figure 2c-e).

To reduce time, we also explored the extension time needed during PCR, where we selected the shortest time without sacrificing complexity or biasing of genes at different transcript lengths (Supplementary Figure 4).

A key concept we devised was to use a low number of amplification cycles to amplify the cDNA libraries. The idea was to remove the redundancy of overamplifying libraries to be able to visualize and quantify them, followed by the need for dilution & normalization of cDNA to save cost on Tn5 enzyme. To explore whether such a strategy was feasible, we systematically investigated how library complexity scaled with a range of varying cDNA input and Tn5 amounts, mimicking a low amplification setting (Figure 1g-k). We observed that library complexity was in general conserved over a surprisingly broad range of cDNA input, which led us to believe that using low amplification cycles was indeed feasible without sacrificing complexity. We then proceeded to show that this still held true over a reasonable range of PCR cycles (Figure 2B). For reference, we typically use 10-12 pre amplification PCR cycles for HEK cell sized cells, and 16 cycles for smaller low content cells like PBMCs. Further optimization may be done by the user, depending on cell samples.

Clean-up & dilution of cDNA: A key change for the reduction in time to prepare Smart-seq3xpress libraries is the omission of QC, clean-up, and normalization of the pre-amplified cDNA before proceeding with tagmentation. Various strategies for cleaning up the preamplified cDNA libraries were tested: (1) Protein removal using ProteinaseK, (2) Primer removal using Exonuclease I (3) dNTP removal using Shrimp Alkaline Phosphatase and derivates (4) clean-up with carboxylated beads and (5) various volumes of H2O dilution (range: 1-20 uL). Overall, we were able to achieve comparable libraries without having to resort to any measure of cleaning up the preamplified libraries other than simple dilution. We decided on a dilution factor of 10, because it yielded both stable and good results, minimal interference with tagmentation and easy dispense using both automated liquid handlers or non-contact dispensers.

Tagmentation & index PCR: Overall, no conceptual changes were applied to the tagmentation procedure. We tested the method both with commercially available and in-house produced Tn5. For commercial Tn5, switching from ATM (Amplicon Tagmentation mix; Nextera XT Kit; Illumina cat. 15032354) to TDE1 (Tagmentation DNA enzyme 1; Illumina cat. 20034198) allowed us to reduce costs drastically, since it seems more concentrated and thus can be used in much lower amounts per cell. This switch did not affect any sequencing metrics in terms of library quality or outcome.

The overall final index PCR volume can be reduced to 5 uL instead of the standard 8 uL, without many problems, making it possible to reduce the cost of this reaction & reducing the volume input into the final library purification. However, this reduction in volumes requires higher accuracy in SDS concentration & dispensing to avoid inhibition of the PCR enzyme, which can be overcome by addition of 0.01-0.05% Tween-20 to the PCR mix.

In order to quickly pool and purify your sequencing ready libraries, we implemented the concept of centrifuging the contents of the PCR plate out into a reservoir, originally presented in Quartz-seq2 (https://genomebiology.biomedcentral.com/articles/10.1186/s13059-018-1407-3). We designed a simple holder that can be 3D printed easily to fit around Nalgene robotic reservoirs (Thermo Scientific cat. 1200-1300) and keeps standard SBS 384-well plates in place. **CAUTION:** We recommend users try out the system with a plate filled with water first before applying it on an actual library. The centrifugation-based pooling requires a swing bucket rotor that can fit the height of the reservoir and plate. The required amount of G force is minimal and quickly pulsing the centrifuge up to 50-100g suffices. **If centrifugation speed is too high, spillage will occur.** Several libraries/plates can be spun into the same reservoir before emptying for easier downstream cleanup, however, overfilling of the reservoir should be avoided. We have evaluated that no more than 3 plates per reservoir should be pooled before emptying.

Sequencing. The produced sequencing libraries are standard Illumina (Nextera-style) compatible. The exact sequencing layout is dependent on what the specific question requires (eg. molecule reconstructions require paired-end sequencing and benefit from longer read lengths, while simple gene expression counting suffices with single-end sequencing). Due to the presence of the 5' recognition sequence, UMI and template-switching G triplet, the minimum supported read length is not below 75 bases. Similarly, sequencing depth is highly project-dependent and the full complexity of libraries is generally not observed with less than 1 million reads. Smart-seq3xpress libraries work equally well on Illumina and MGI sequencers.

Note References:

1. Tang, D. T. P. *et al.* Suppression of artifacts and barcode bias in high-throughput transcriptome analyses utilizing template switching. *Nucleic Acids Res.* **41**, e44 (2013).
2. Hahaut, V., Pavlinic, D., Cowan, C. & Picelli, S. *Lightning Fast and Highly Sensitive Full-Length Single-cell sequencing using FLASH-Seq*. 2021.07.14.452217 https://www.biorxiv.org/content/10.1101/2021.07.14.452217v1 (2021) doi:10.1101/2021.07.14.452217.
3. Hagemann-Jensen, M. *et al.* Single-cell RNA counting at allele and isoform resolution using Smart-seq3. *Nat. Biotechnol.* **38**, 708–714 (2020).
4. Ziegenhain, C., Hendriks, G.-J., Hagemann-Jensen, M. & Sandberg, R. *Molecular spikes: a gold standard for single-cell RNA counting*. 2021.07.10.451877

https://www.biorxiv.org/content/10.1101/2021.07.10.451877v1 (2021)

doi:10.1101/2021.07.10.451877.
