# Supplementary material for: Scalable single-cell RNA sequencing from full transcripts with Smart-seq3xpress
Source: Nat Biotechnol. 2022 May 30;40(10):1452–7. doi: 10.1038/s41587-022-01311-4 (PMC9546772; doi:10.1038/s41587-022-01311-4)
Supplement: Supplementary file 2 — Reporting Summary [file 41587_2022_1311_MOESM2_ESM.pdf]

Corresponding author(s): Rickard Sandberg

Last updated by author(s): Mar 23, 2022

Reporting Summary

Nature Research wishes to improve the reproducibility of the work that we publish. This form provides structure for consistency and transparency in reporting. For further information on Nature Research policies, see our Editorial Policies and the Editorial Policy Checklist.

Statistics

For all statistical analyses, confirm that the following items are present in the figure legend, table legend, main text, or Methods section.

n/a	Confirmed	
☐	☒	The exact sample size (*n*) for each experimental group/condition, given as a discrete number and unit of measurement
☐	☒	A statement on whether measurements were taken from distinct samples or whether the same sample was measured repeatedly
☐	☒	The statistical test(s) used AND whether they are one- or two-sided<br>*Only common tests should be described solely by name; describe more complex techniques in the Methods section.*
☐	☒	A description of all covariates tested
☐	☒	A description of any assumptions or corrections, such as tests of normality and adjustment for multiple comparisons
☐	☒	A full description of the statistical parameters including central tendency (e.g. means) or other basic estimates (e.g. regression coefficient) AND variation (e.g. standard deviation) or associated estimates of uncertainty (e.g. confidence intervals)
☐	☒	For null hypothesis testing, the test statistic (e.g. *F*, *t*, *r*) with confidence intervals, effect sizes, degrees of freedom and *P* value noted<br>*Give P values as exact values whenever suitable.*
☐	☒	For Bayesian analysis, information on the choice of priors and Markov chain Monte Carlo settings
☒	☐	For hierarchical and complex designs, identification of the appropriate level for tests and full reporting of outcomes
☐	☒	Estimates of effect sizes (e.g. Cohen's *d*, Pearson's *r*), indicating how they were calculated

Our web collection on statistics for biologists contains articles on many of the points above.

Software and code

Policy information about availability of computer code

Data collection	The following software was used in data collection: FACS sorter software (BD FACSChorus 1.3), sequencer software (Illumina NextSeq Control Software 2.2.0 and MGI DNBSEQ G400RS 1.1.0.108).
Data analysis	Analysis was performed using zUMIs v2.8.2 or zUMIs v2.9.3f, STAR v2.7.3a, Seurat v4.0.1, presto (v1.0.0), Cellbender, Solo v0.6, TRaCeR v0.6.0, Rsamtools v2.6.0, UMIcountR v0.1.1, cellSNP-lite v1.0.0, BRIEkit v0.2.2, BRIE2 v2.0.6., CellBender v0.2.0,BSgenome v1.62.0, Scanpy v1.8.2, Scirpy v0.8.0, Gviz v1.38.1L, rtracklayer v1.54.0, Further details are listed in the Methods section.<br>zUMIs is available at https://github.com/sdparekh/zUMIs. Code to filter reads with the TSO strand invasion artifact is implemented in a stand-alone script pyTSOfilter (https://github.com/cziegenhain/pyTSOfilter).

For manuscripts utilizing custom algorithms or software that are central to the research but not yet described in published literature, software must be made available to editors and reviewers. We strongly encourage code deposition in a community repository (e.g. GitHub). See the Nature Research guidelines for submitting code & software for further information.

Data

Policy information about availability of data

All manuscripts must include a data availability statement. This statement should provide the following information, where applicable:

- Accession codes, unique identifiers, or web links for publicly available datasets
- A list of figures that have associated raw data
- A description of any restrictions on data availability

Sequencing data have been deposited at ArrayExpress, European Bioinformatics Institute, under the following accession numbers; E-MTAB-11488, E-MTAB-11452, E-MTAB-11467. Human genome build hg38 fasta files and gene annotation in GTF format (Grch38.95) were obtained from Ensembl.

Field-specific reporting

Please select the one below that is the best fit for your research. If you are not sure, read the appropriate sections before making your selection.

☒ Life sciences ☐ Behavioural & social sciences ☐ Ecological, evolutionary & environmental sciences

For a reference copy of the document with all sections, see nature.com/documents/nr-reporting-summary-flat.pdf

Life sciences study design

All studies must disclose on these points even when the disclosure is negative.

Sample size	Sample sizes were not predetermined using statistical analysis. They were determined based on allowable size within a reasonable budget cost for preparing libraries and sequencing
Data exclusions	Single-cell RNA-seq data were filtered according to established criteria. Cutoffs are listed where appropriate. Further data exclusions were not performed.
Replication	For each experimental condition, a large number of single cells was sequenced to ensure reprodocibility. Sample sizes are clearly indicated throughout.
Randomization	Not relevant because FACS sorting of individual cells into random wells of microplates.
Blinding	Investigators were not blinded to groups of samples as it was not practically feasible.

Reporting for specific materials, systems and methods

We require information from authors about some types of materials, experimental systems and methods used in many studies. Here, indicate whether each material, system or method listed is relevant to your study. If you are not sure if a list item applies to your research, read the appropriate section before selecting a response.

Materials & experimental systems

n/a	Involved in the study	
☐	☒	Antibodies
☐	☒	Eukaryotic cell lines
☒	☐	Palaeontology and archaeology
☒	☐	Animals and other organisms
☐	☒	Human research participants
☒	☐	Clinical data
☒	☐	Dual use research of concern

Methods

n/a	Involved in the study	
☒	☐	ChIP-seq
☒	☐	Flow cytometry
☒	☐	MRI-based neuroimaging

Antibodies

Antibodies used	All antibodies used are from BD Bioscience (Pharmingen & Horizon). PE Mouse Anti-Human CCR7 (Cat.no: 566742, Clone: 2-L1-A, Lot:1006133), PE-Cy7 Mouse Anti-Human CD4 (Cat. no: 557852 Clone:SK3, Lot: 3060697), FITC Mouse Anti-Human CD45RA (Cat. no: 561882, Clone:HI100, Lot: 9301282), PerCP-Cy5.5 / BB700 Mouse Anti-Human CD8 (Cat. no: 566451, Clone:RPA-T8, Lot: 1011278), PE-Cy5 Mouse Anti-Human CD45RO (Cat.no: 561888, Clone:UCHL1, Lot: 9101742)
Validation	All antibodies are validated for reactivity against Human, and applicable to FACS sorting according to manufacturer BD Bioscience.

Eukaryotic cell lines

Policy information about cell lines

Cell line source(s)	HEK293FT: Thermo Fisher, K562: DSMZ (Braunschweig, Germany)
Authentication	HEK293FT and K562 were authenticated by PCR-single-locus-technology (Eurofins Forensik)
Mycoplasma contamination	HEKF293FT and K562 were confirmed free of mycoplasma contamination (Eurofins)
Commonly misidentified lines (See ICLAC register)	HEK293FT and K562 were used but authenticity was confirmed, both are not part of common misidentified cell lines (see above).

Human research participants

Policy information about studies involving human research participants

Population characteristics	No specific population selections or criterias were used, other than overall healthy donors. The 7 healthy donors, include 4 male, 2 female, 1 N/A. Age range 21-47
Recruitment	Frozen Aliquots of cryopreserved human PBMCs were bought from Lonza.
Ethics oversight	Etikprövningsmyndigheten (Sweden) Dnr 2020-05070

Note that full information on the approval of the study protocol must also be provided in the manuscript.
